# Adjunctive Strategies to Enhance Antimicrobial Photodynamic Therapy in Oral Infections: A Systematic Review of Randomized Controlled Trials

**DOI:** 10.3390/jcm15207959

**Published:** 2026-10-14

**Authors:** Aleksandra Warakomska, Małgorzata Kępa, Martin Starosta, Barbara Lipka, Hanna Hüpsch-Marzec, Dariusz Skaba, Rafał Wiench

**Affiliations:** 1 Department of Periodontal and Oral Mucosa Diseases, Faculty of Medical Sciences in Zabrze, Medical University of Silesia, 40-055 Katowice, Poland; awarakomska@sum.edu.pl (A.W.); hanna.hupsch-marzec@sum.edu.pl (H.H.-M.); 2 Department of Microbiology, Faculty of Pharmaceutical Sciences in Sosnowiec, Medical University of Silesia in Katowice, Jagiellońska 4, 41-200 Sosnowiec, Poland; mkepa@sum.edu.pl; 3 Department of Dentistry, Faculty of Medicine, University of Ostrava, Syllabova 19, Ostrava-Jih, 703 00 Ostrava, Czech Republic; martin.starosta@osu.cz

**Keywords:** antimicrobial photodynamic therapy, oral biofilm, periodontitis, peri-implantitis, photosensitizer, randomized controlled trial, adjunctive therapy

## Abstract

**Background/Objectives:** Antimicrobial photodynamic therapy (aPDT) is a widely investigated adjunct for managing oral biofilm-associated bacterial and fungal infections, but poor photosensitizer penetration, aggregation, biofilm hypoxia, and protocol variability limit its clinical efficacy. **Methods**: This review was registered with PROSPERO (CRD420251207522) and conducted according to PRISMA 2020 guidelines. PubMed/MEDLINE, Embase, Scopus, and the Cochrane Library were searched for English-language RCTs. Eligible studies evaluated aPDT combined with physicochemical, delivery-based, light-based, biological, or host-modulating adjuncts in patients with oral biofilm-related bacterial or fungal disease, using an appropriate comparator. Data were synthesized narratively due to substantial clinical and methodological heterogeneity, and risk of bias was assessed using RoB 2. **Results:** Fourteen RCTs were included, predominantly addressing periodontal and peri-implant conditions, with additional studies involving orthodontic biofilms; no eligible RCT evaluated a fungal oral infection. Surfactant-based enhancement (e.g., sodium dodecyl sulfate) produced formulation- and protocol-dependent effects, with benefits reported for methylene blue and curcumin but not riboflavin. Nanocarrier-based photosensitizer delivery, evaluated in combination with *Aloe vera* vehicles, improved selected clinical outcomes without consistently superior microbiological effects; however, the independent contribution of the nanocarrier could not be isolated from the potential bioactive effects of the vehicle. An oxygen-releasing adjunct showed no significant between-group clinical benefit. Repeated dual-light irradiation improved bleeding on probing and pocket-related outcomes in some but not all trials. Probiotics, vitamin D, and low-level laser therapy showed inconsistent or preliminary benefits, each supported by limited trial numbers. Risk-of-bias assessments predominantly indicated low risk or some concerns, with one study judged at high overall risk. **Conclusions:** Enhancement of aPDT should not be regarded as a single therapeutic concept; its clinical benefit is strategy-, formulation-, and context-dependent. Current randomized evidence does not support a universally superior adjunct, highlighting the need for standardized, adequately powered trials with mechanism-oriented comparator designs.

## 1. Introduction

### 1.1. Rationale

Oral infectious diseases pose a significant challenge in contemporary dentistry. They encompass a broad range of conditions, including periodontal and peri-implant diseases, endodontic infections, dental caries, and yeast infections. The primary etiological factor underlying these diseases is the formation of microbial biofilms, which are highly resistant to host immune responses and conventional antimicrobial therapies [1,2]. Their persistence promotes chronic inflammation, tissue destruction, disease recurrence, and treatment failure, highlighting the need for more effective adjunctive therapeutic strategies [2,3,4]. Conventional management of oral infections primarily relies on mechanical debridement, supplemented by topical antiseptics or systemic antibiotic therapy [2,5]. Although mechanical methods are essential for disrupting biofilms, they often fail to eradicate microorganisms from anatomically complex sites, such as periodontal pockets, root canal systems, implant surfaces, and dentinal tubules [4,5,6]. Adjunctive antiseptics and systemic antibiotics may improve short-term clinical outcomes. Still, their effectiveness is limited by poor penetration into mature biofilms, potential adverse effects, and the growing prevalence of antimicrobial resistance [1,4,7]. These challenges have prompted the exploration of alternative, non-antibiotic antimicrobial strategies. Antimicrobial photodynamic therapy (aPDT) has emerged as a promising adjunctive modality for the management of oral infections [8,9]. aPDT uses a photosensitizer, a specific wavelength of light, and molecular oxygen to generate reactive oxygen species that induce rapid microbial cell death [8,9,10,11]. Although conventional microbial resistance to aPDT is considered relatively uncommon due to its multi-targeted mode of action, repeated sublethal exposure may induce adaptive responses or increased microbial tolerance [12]. Consequently, numerous experimental, preclinical, and clinical studies have demonstrated the antimicrobial efficacy of aPDT against a wide spectrum of oral pathogens, including bacteria and fungi, in both planktonic and biofilm states [5,13,14,15].

Despite these advantages, several biological and physicochemical limitations constrain the clinical effectiveness of aPDT. These include inadequate penetration of photosensitizers into mature biofilms, photosensitizer aggregation and instability, oxygen depletion in hypoxic biofilm environments, and variability in treatment protocols across irradiation parameters and photosensitizer formulations [10,16]. Recent research has therefore increasingly focused on adjunctive enhancement strategies to improve the efficacy of aPDT. Examples include incorporating photosensitizers into nanocarriers; using surfactants, polymers, cyclodextrins, and inorganic salts; and employing oxygen-delivery systems and multifunctional hybrid platforms to improve photosensitizer delivery, biofilm penetration, and reactive oxygen species generation [10,16,17].

Given the rapidly expanding and methodologically diverse literature in this field, a comprehensive and critical synthesis of the available evidence is necessary. To our knowledge, no previous systematic review has evaluated adjunctive aPDT enhancement strategies across different oral infectious diseases while focusing exclusively on randomized controlled clinical trials. Previous reviews have addressed individual enhancement approaches or specific oral conditions, whereas the present review integrates multiple physicochemical, delivery-based, light-based, biological, and host-modulating strategies within a single clinical evidence framework [18,19,20,21]. Because these strategies address different limitations of conventional aPDT through distinct mechanisms, their effects should be considered individually rather than treating enhanced aPDT as a single therapeutic approach. This systematic review aims to critically appraise and synthesize current evidence on adjunctive approaches that potentiate aPDT against oral biofilm-associated bacterial and fungal infections, with the specific aim of evaluating their effects on clinical outcomes and informing the optimization of treatment protocols and future translational and clinical research.

### 1.2. Objectives

The primary objective of this systematic review was to critically evaluate the clinical effectiveness of adjunctive strategies designed either to enhance the photodynamic process or to complement the therapeutic antimicrobial effect of aPDT-based interventions in the management of oral biofilm-associated bacterial and fungal conditions. Specifically, the review sought to determine whether incorporation of such strategies provides additional clinical and/or antimicrobial benefits compared with conventional aPDT or other appropriate control interventions.

The secondary objectives were to identify and compare the various enhancement strategies investigated in randomized clinical trials and to assess their effects on clinically relevant outcomes, including microbial reduction, biofilm control, clinical improvement, and treatment safety. Particular emphasis was placed on evaluating the consistency of treatment effects across different oral biofilm-associated conditions represented in the eligible randomized clinical evidence.

By systematically synthesizing evidence from randomized clinical trials, this review aims to provide an evidence-based assessment of the clinical value of enhanced aPDT protocols, support the optimization of treatment strategies, and identify priorities for future clinical research.

## 2. Materials and Methods

### 2.1. Focused Question

This systematic review was conducted according to the PICO framework to address the following focused question: In patients with oral biofilm-related bacterial or fungal diseases (Population), does antimicrobial photodynamic therapy (aPDT) combined with adjunctive enhancement strategies (Intervention), compared with conventional aPDT without adjunctive enhancement or other eligible control interventions (Comparison), improve clinical and/or microbiological outcomes (Outcome) [22]? The review was restricted to randomized controlled trials.

### 2.2. Search Strategy

This systematic review was registered with PROSPERO under the registration number CRD420251207522. It was conducted and reported in accordance with the PRISMA 2020 (Preferred Reporting Items for Systematic Reviews and Meta-Analyses) statement [23]. A comprehensive literature search was conducted in PubMed/MEDLINE, Embase, Scopus, and the Cochrane Library using predefined search strategies (Table 1). The search was restricted to English-language articles published between 9 November 2015 and 9 November 2025. This ten-year window was selected a priori to capture the contemporary generation of aPDT enhancement technologies, photosensitizer formulations, and irradiation protocols relevant to current clinical practice, and to keep the scope of a first systematic synthesis of this rapidly evolving field feasible; we did not search for records before 2015, as we decided to limit the search as per PRISMA guidelines. All four databases were last searched on 9 November 2025. No deviations from the registered PROSPERO protocol occurred during the conduct of this review.

**Figure 1:**
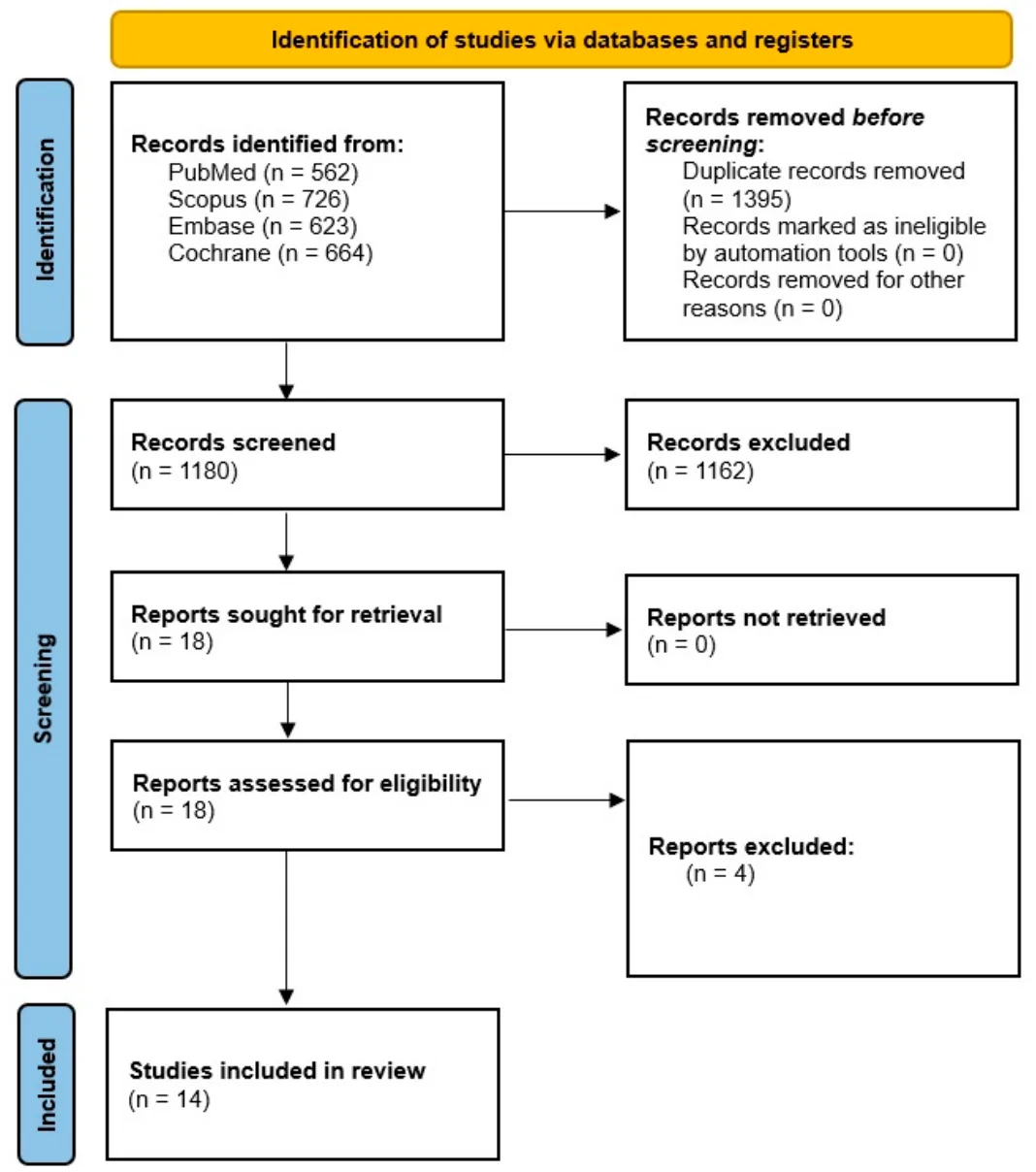
PRISMA 2020 flow diagram.

The reference lists of the included articles were additionally screened to identify potentially relevant studies not retrieved through the electronic database searches. The study identification and selection process is presented in Figure 1. All 18 reports sought for retrieval were successfully obtained and assessed for eligibility. Following full-text assessment, four reports were excluded because they did not meet the predefined eligibility criteria regarding the intervention and/or comparator, resulting in 14 studies being included in the review.

Following duplicate removal, the titles and abstracts of all retrieved records were independently screened by two reviewers for potential eligibility according to the predefined criteria outlined in Table 2, and disagreements were resolved through discussion and, where necessary, consultation with a third reviewer. Records considered potentially relevant, or for which eligibility could not be reliably determined from the title and abstract alone, were retained for full-text assessment. Full-text reports were subsequently evaluated independently by the same two reviewers against the predefined eligibility criteria, with disagreements again resolved by discussion or third-reviewer adjudication.

### 2.3. Study Selection and Eligibility Criteria

Randomized controlled trials were eligible if they investigated aPDT incorporating or combined with an adjunctive strategy intended either to enhance the photodynamic process or to complement the therapeutic antimicrobial effect of an aPDT-based intervention in the management of oral biofilm-associated bacterial or fungal conditions. Eligible strategies included physicochemical, delivery-based, light-based, biological, and host-modulating approaches acting either directly on components of the photodynamic process or as complementary adjuncts to an aPDT-containing treatment protocol. For clarity, strategies that act directly on the photodynamic process itself—namely surfactant/permeabilizing agents, nanocarrier-based delivery systems, and oxygen-releasing or oxygen-generating adjuncts—were considered direct aPDT enhancers, whereas strategies with a primarily biological or host-modulating mode of action—namely probiotics, vitamin D supplementation, and low-level laser therapy—were considered complementary adjuncts added to an aPDT-containing protocol rather than direct modifiers of the photodynamic reaction; this distinction is used throughout the Results and Discussion.

Studies were required to include an appropriate control or comparison condition permitting evaluation of the enhanced or complementary aPDT-based intervention. For complementary adjuncts, the study design was required to permit assessment of the adjunct’s contribution relative to an otherwise comparable aPDT-containing treatment protocol. Strategies incorporated directly into the experimental aPDT protocol were eligible when the comparator permitted evaluation of the resulting modified aPDT-based intervention, even when the individual contribution of each component could not be isolated. Accordingly, the included trials are best understood as falling into two comparator strata that answer different questions and should not be interpreted as providing equivalent evidence for “enhancement”: (i) trials comparing an enhanced aPDT protocol against conventional, unenhanced aPDT (e.g., SDS-containing versus aqueous photosensitizer formulations), which isolate the contribution of the enhancement itself; and (ii) trials comparing an aPDT-containing treatment protocol (enhanced or conventional) against standard non-aPDT therapy, which evaluate the contribution of aPDT (with or without enhancement) as a whole rather than the enhancement component in isolation. This stratification is applied when interpreting individual study findings in Section 3 and Section 4.

Studies were excluded if they did not investigate an oral biofilm-associated bacterial or fungal condition, did not employ a randomized controlled design, evaluated photodynamic therapy for purposes other than antimicrobial treatment, or investigated conventional aPDT without an eligible enhancement or complementary strategy. Studies evaluating complementary adjuncts were also excluded when the comparator did not permit assessment of their contribution within an aPDT-containing treatment protocol. Additional antimicrobial or supportive interventions administered solely as independent concomitant treatments, without a defined role in enhancing or complementing the aPDT-based intervention, were considered ineligible. Non-randomized interventional studies, observational studies, case reports, case series, reviews, and other secondary research were excluded. Conference abstracts, trial registry records, protocols, and other supplementary reports were not considered independent eligible studies. However, when linked to an eligible randomized controlled trial, supplementary reports could be retained and, where appropriate, used to verify study characteristics and methodological information.

### 2.4. Data Extraction

Following the final selection of eligible studies, data were independently extracted by two reviewers using a standardized data extraction form. Any discrepancies were resolved through discussion and consensus. Extracted study characteristics included the first author, year of publication, country, study design, sample size, intervention and comparator groups, target oral biofilm-related disease, and duration of follow-up.

Detailed information regarding the aPDT protocols was also collected, including the photosensitizer, its concentration, light source, wavelength, irradiation mode, power output, irradiation time, and, when reported, pre-irradiation/dark incubation time, spot or fiber area, radiant exposure/fluence, total delivered energy, irradiation geometry, the number of treated sites, the number and frequency of treatment sessions, and whether irradiation was applied in contact or non-contact mode, as these variables materially affect microbial photoinactivation.

Particular attention was given to the adjunctive strategy used in combination with aPDT. For each study, the type of adjunct, mode of application, and proposed mechanism of action were recorded. Enhancement strategies were categorized according to their principal role within the aPDT-based intervention as physicochemical, delivery-based, light-based, biological, or host-modulating approaches, and, as noted in Section 2.3, further distinguished as direct enhancers of the photodynamic process or complementary adjuncts to an aPDT-containing protocol.

Extracted outcomes included microbiological, clinical, and patient-reported measures, where available. For microbiological outcomes, extraction captured, where reported, the sample type and anatomical site, sampling time point relative to treatment, analytical method (e.g., culture/CFU counting, quantitative PCR, confocal laser scanning microscopy [CLSM]), the target microorganism(s), the units of measurement, and whether the endpoint represented viable cell counts or total microbial DNA/biomass. These forms of “microbial burden or viability” are not interchangeable and were recorded separately whenever the primary studies allowed this distinction to be made. Clinical outcomes included disease-specific parameters such as bleeding on probing (BoP), probing depth (PD), plaque index (PI), clinical attachment level (CAL), marginal bone level (MBL), and other relevant clinical measures. Adverse events and safety-related outcomes were also extracted when reported, together with information relevant to the safety of the adjunctive strategies.

### 2.5. Data Synthesis

Owing to substantial clinical and methodological heterogeneity across the included studies, particularly in the oral conditions investigated, enhancement strategies, aPDT protocols, comparators, follow-up periods, and outcome definitions and measurement time points, quantitative pooling was deemed inappropriate. This judgment was based on the following predefined considerations: (i) fewer than two studies shared an identical enhancement strategy, photosensitizer, comparator type, and outcome definition at a comparable follow-up time point, which is the minimum configuration required for a clinically interpretable pairwise meta-analysis; (ii) even within the same enhancement category (e.g., dual-light irradiation or nanocarrier-based delivery), studies differed in comparator stratum (enhanced-vs-conventional aPDT versus aPDT-containing-vs-standard-therapy, see Section 2.3), precluding pooling of effect estimates that answer different clinical questions; and (iii) outcome measurement instruments and time points were not sufficiently aligned across studies investigating the same enhancement category to support a clinically meaningful pooled estimate. The findings were therefore synthesized narratively, with studies considered according to the principal role of the enhancement strategy within the aPDT-based intervention to facilitate comparison of their characteristics, therapeutic effects, and current evidence gaps.

### 2.6. Risk of Bias Assessment

The risk of bias in the included randomized controlled trials was independently assessed by two reviewers using the Revised Cochrane Risk of Bias tool (RoB 2). Disagreements between the reviewers were resolved through discussion and consensus.

Risk-of-bias assessments were performed for outcomes considered relevant to the objectives of the present review, with primary outcomes prioritized when clearly prespecified by the study authors. When no primary outcome was explicitly defined, a clinically or microbiologically relevant outcome that most closely aligned with the review’s objectives was selected for assessment. Because RoB 2 judgments are specific to the evaluated outcome and its assessment time point rather than to the study as a whole, the specific outcome and follow-up time point assessed for each trial are reported alongside the domain-level judgments in Table 3.

The RoB 2 tool evaluates five domains: (1) bias arising from the randomization process; (2) bias due to deviations from intended interventions; (3) bias due to missing outcome data; (4) bias in the measurement of the outcome; and (5) bias in the selection of the reported result. Each domain was evaluated using the RoB 2 signaling questions and was judged as having a low risk of bias, some concerns, or a high risk of bias. An overall risk-of-bias judgment—low risk, some concerns, or high risk, in accordance with RoB 2 guidance, which does not include a “moderate” risk category—was subsequently assigned to each evaluated result. The risk-of-bias assessments for the included studies are presented in Table 3.

## 3. Results

### 3.1. Characteristics of the Included Studies

The characteristics of the included randomized controlled trials are summarized in Table 4, Table 5, Table 6 and Table 7. The studies investigated a range of strategies intended to enhance or complement antimicrobial photodynamic therapy (aPDT) in the management of oral biofilm-related conditions, predominantly periodontitis, but also peri-implant diseases and orthodontic biofilms; no eligible randomized trial addressing an oral fungal biofilm-associated condition (e.g., oral candidiasis) was identified. The included studies varied considerably in study population, intervention design, aPDT protocol, and the type of enhancement strategy evaluated.

Study groups and the principal clinical and microbiological outcomes are summarized in Table 4, which also reports, where available, the adverse events and safety-related findings for each trial. Technical characteristics of the aPDT protocols, including the photosensitizer, photosensitizer concentration, light source, wavelength, and irradiation parameters, are presented in Table 5. The adjunctive enhancement strategies, their classification, and proposed mechanisms of action are summarized in Table 6. The distribution of the included studies according to dental field and biofilm-related condition is presented in Table 7. Owing to substantial heterogeneity across studies in terms of clinical conditions, enhancement strategies, aPDT protocols, follow-up periods, and reported outcome measures, the findings were synthesized qualitatively.

### 3.2. Overview of Study Characteristics

The included trials were conducted across several geographical regions, including Europe, Asia, South America, and the Middle East, reflecting considerable geographical diversity in the available evidence. The countries in which the individual studies were clinically conducted are presented in Table 8. The included studies varied considerably in sample size and clinical setting.

Most studies focused on periodontal diseases, including different stages and severities of periodontitis, while others investigated peri-implant conditions or orthodontic appliance-associated biofilms (Table 7); no eligible trial addressed an oral fungal biofilm-associated condition. Despite differences in study populations and clinical indications, the included trials evaluated strategies to enhance or complement the therapeutic effects of aPDT.

Considerable methodological heterogeneity was observed in the aPDT protocols. The photosensitizers included methylene blue, toluidine blue, curcumin, riboflavin, and indocyanine green, while light sources and irradiation parameters varied substantially across studies. The investigated enhancement approaches included physicochemical strategies such as surfactants, nanocarrier-based systems, and oxygen-enhancing approaches; biological strategies, including probiotics and host-modulating supplementation; and physical approaches, such as dual-light irradiation or photobiomodulation. This diversity illustrates the range of approaches investigated to optimize or complement aPDT through different physicochemical, biological, and physical mechanisms.

### 3.3. Main Study Outcomes

Overall, the included studies demonstrated heterogeneous effects of adjunctive strategies on the clinical and antimicrobial efficacy of antimicrobial photodynamic therapy. Several enhancement approaches were associated with additional benefits compared with conventional aPDT or standard treatment alone, although these effects were not consistent across all studies or outcome measures. Reported benefits included reductions in microbial load and improvements in clinical periodontal parameters, particularly bleeding on probing (BoP), probing depth (PD/PPD), and clinical attachment level (CAL). Where relevant, the findings below distinguish immediate photoinactivation effects (e.g., CFU reduction assessed shortly after irradiation) from longer-term microbiological or clinical outcomes assessed at 3–6 months, since the latter are influenced by recolonization, oral hygiene, and host factors in addition to any direct photodynamic effect, and treat CFU counts, species-specific quantitative PCR/culture counts, and CLSM-based biofilm-viability imaging as distinct, non-interchangeable measures of antimicrobial efficacy.

Surfactant-based enhancement produced variable antimicrobial effects across the included studies. Alvarenga et al. (2019) [24] reported that aqueous methylene blue (MB)-mediated aPDT did not significantly reduce bacterial survival immediately after treatment, regardless of irradiation time. In contrast, the addition of sodium dodecyl sulfate (SDS) was associated with a significant reduction in immediate bacterial survival after 5 min of irradiation; however, because the SDS-containing and aqueous MB formulations were assigned to two sequential, non-randomized cohorts rather than randomized within the same cohort (Table 4), this finding should be interpreted as hypothesis-generating rather than as a randomized comparison of the SDS formulation itself. Shorter irradiation times with the surfactant-containing formulation did not yield significant reductions, suggesting that the observed enhancement depended on irradiation duration. Kassa et al. (2022) [25] similarly observed lower immediate bacterial survival and reduced colony growth following aPDT with MB diluted in SDS compared with the other treatment conditions. However, although the overall difference among groups was significant, post-hoc comparisons did not identify significant differences between individual groups, limiting evidence for a definitive additional antimicrobial effect of SDS.

Panhóca et al. (2016) [29] found that both curcumin-mediated PDT and curcumin combined with SDS significantly reduced the viability of salivary *S. mutans*. The surfactant-enhanced protocol produced a greater numerical reduction in salivary bacterial counts than curcumin-mediated PDT alone, and its antimicrobial effect did not differ significantly from that of chlorhexidine; because outcomes were measured in saliva rather than at the treated biofilm site, these findings characterize whole-mouth microbial load rather than site-specific biofilm eradication. In contrast, Sudhakar et al. (2025) [32] found no additional CFU-based antimicrobial benefit from incorporating SDS into riboflavin-mediated aPDT, using plaque samples collected directly from the treated site. For *S. mutans*, riboflavin-mediated aPDT alone resulted in significantly lower immediate bacterial counts than the riboflavin-SDS protocol; however, this difference was no longer significant at the 2-month follow-up. For *L. acidophilus*, the riboflavin-SDS protocol reduced bacterial counts compared with the chlorhexidine control, but no significant differences were observed between riboflavin-mediated aPDT with and without SDS. CLSM biofilm-viability imaging—a distinct measure from CFU counting—further showed the lowest residual biofilm viability with riboflavin-mediated aPDT alone and the highest with the riboflavin-SDS protocol.

Nanocarrier- and bioactive vehicle-based approaches showed favorable clinical effects in periodontal and peri-implant applications, assessed over 3–6 months of follow-up rather than as an immediate photoinactivation endpoint. Qamar et al. (2023) [26] reported that riboflavin-loaded PGA nanoparticles incorporated into *Aloe vera* gel and combined with mechanical debridement resulted in significantly greater improvements in plaque index, probing depth, and marginal bone level than conventional riboflavin-mediated aPDT or mechanical debridement alone. However, conventional aPDT produced the greatest overall reduction in peri-implant microbial counts, including significant reductions in *P. gingivalis* and *T. forsythia* during follow-up. Elsadek et al. (2023) [33] similarly found that methylene blue-loaded PGA nanoparticles incorporated into *Aloe vera* gel and combined with SRP resulted in significantly greater probing depth reduction and CAL gain than conventional methylene blue-mediated aPDT or SRP alone. In contrast, conventional aPDT produced a greater reduction in BoP. Microbiological findings were less consistent: conventional aPDT reduced both *T. forsythia* and *P. gingivalis* at 3 months, whereas the nanoparticle-based formulation was associated with a significant reduction in *T. forsythia* only. In both trials, the nanoparticle and *Aloe vera* gel components were combined without a comparator arm isolating either component, so the relative contribution of the nanocarrier versus the bioactive *Aloe vera* vehicle to the observed clinical effects cannot be determined from these data (see the “Nanocarriers” subsection in Section 4.1.2).

An oxygen-based microenvironmental enhancement strategy was evaluated by Cláudio et al. (2024) [28] in patients with poorly controlled type 2 diabetes and residual periodontal pockets. Because this trial did not include a conventional aPDT-only arm, its findings are reported here as effects of the combined oxygen-releasing gel + aPDT protocol rather than as evidence that oxygen supplementation specifically enhanced aPDT. The oxygen-releasing gel combined with aPDT produced a significant within-group reduction in plaque index at 90 days and reduced the proportion of sites with PD ≥5 mm and BoP at 90 and 180 days compared with baseline. However, no statistically significant between-group differences were detected for the evaluated clinical periodontal parameters. Significant within-group reductions in PD and CAL were also observed following subgingival instrumentation alone and oxygen-releasing gel without aPDT, indicating that the additional clinical contribution of the combined oxygen-releasing gel-aPDT protocol could not be clearly isolated. A transient reduction in TNF-α was observed in the oxygen-releasing gel + aPDT group at 90 days, whereas no significant between-group differences were observed in the evaluated immunological outcomes.

Biological adjunctive strategies based on probiotic supplementation produced inconsistent additional benefits. Mongardini et al. (2017) [27] found that adjunctive probiotics did not confer additional clinical benefits when combined with mechanical plaque removal and photodynamic therapy in an experimental model of peri-implant mucositis. Both probiotic and placebo interventions significantly reduced plaque accumulation during follow-up, with no significant between-treatment differences in plaque levels or the number of BoP-positive peri-implant sites.

In contrast, Patyna et al. (2021) [34] reported additional benefits from probiotic supplementation when combined with subgingival debridement and light-activated disinfection in patients with periodontitis. Although PPD and CAL improved in all treatment groups without significant between-group differences, the addition of probiotics resulted in significantly greater reductions in BoP at 3 and 6 months and lower gingival inflammation compared with light-activated disinfection without probiotics at 6 months. The probiotic-containing protocol also produced significantly greater reductions in *P. gingivalis* and *T. forsythia* at 6 months, as assessed by species-specific quantitative methods. In contrast, no significant between-group differences were observed for the remaining periodontal pathogens investigated.

A distinct host-modulating approach was evaluated by El Refai et al. (2025) [36]. The addition of oral vitamin D3 supplementation to an otherwise identical NSPT and methylene blue-mediated PDT protocol resulted in greater short-term improvements in periodontal clinical parameters. CAL improved significantly over time only in the vitamin D group, with a significant between-group difference emerging at 6 weeks. Plaque and gingival indices decreased in both groups, but the reductions were significantly greater with adjunctive vitamin D supplementation at 4 and 6 weeks.

Light-based enhancement strategies were predominantly investigated using adjunctive home-use dual-light aPDT, applied repeatedly over weeks to months rather than as a single-session photoinactivation protocol. Vakaki et al. (2025) [30] found that both NSPT alone and NSPT combined with daily dual-light aPDT significantly improved BoP, PD, and CAL in smokers with Stage III-IV periodontitis. Although a greater short-term reduction in BoP was observed with dual-light aPDT at 2 weeks, no significant between-group differences in the principal clinical periodontal outcomes were demonstrated during subsequent follow-up. Changes in aMMP-8 levels were also not statistically significant. In contrast, Pakarinen et al. (2022) [31] reported additional clinical benefits of home-use dual-light aPDT at the 3-month interim analysis. Adjunctive dual-light treatment resulted in a greater reduction in BoP than NSPT alone and increased the proportion of patients achieving the periodontal health target of BoP < 10%. A greater proportion of patients receiving dual-light aPDT also achieved reductions in the number of deep periodontal pockets, including ≥50% reductions. In contrast, plaque control improved only in the dual-light group. Lähteenmäki et al. (2025) [35] evaluated the same dual-light approach in patients with peri-implant disease and observed a greater reduction in peri-implant inflammation with adjunctive home-use treatment. BoP and aMMP-8 levels decreased in the dual-light group, whereas plaque levels improved in both groups. Reductions in peri-implant pocket depth were observed in some patients; however, no significant between-group differences in pocket depth were demonstrated. A distinct light-based enhancement strategy involving adjunctive low-level laser therapy (LLLT) was evaluated by Malgikar et al. (2016) [37]. All treatment modalities improved periodontal parameters during follow-up; however, the combination of SRP, methylene blue-mediated PDT, and LLLT produced the greatest reductions in PD and gains in CAL. Inter-group comparisons demonstrated significantly greater PD reduction with adjunctive LLLT than with SRP and PDT alone at 3 and 6 months. Improvements in plaque and gingival indices also favored the combined PDT-LLLT protocol at selected follow-up time points. No postoperative pain, discomfort, or treatment-related complications were reported.

Overall, the available evidence suggests that the effectiveness of aPDT enhancement is strategy- and context-dependent, with substantial variability according to the type of adjunct, photosensitizer, irradiation protocol, clinical indication, and evaluated outcome. Given the methodological and clinical heterogeneity of the included studies, direct comparisons between individual enhancement strategies should be interpreted with caution.

### 3.4. Characteristics of Irradiation Protocols

Considerable variation was observed in irradiation parameters among the included studies (Table 5). Both diode laser- and LED-based light sources were employed, with wavelengths ranging from 385–515 nm for broad-spectrum LED irradiation to 980 nm for diode laser irradiation. In most studies, the selected wavelengths corresponded to the conventional principal absorption regions of the respective photosensitizers, including red-light activation of methylene blue and toluidine blue, blue-light activation of curcumin, and near-infrared irradiation of indocyanine green. However, as noted in Table 5, three studies reported photosensitizer-wavelength combinations (riboflavin at 670 nm; methylene blue at 470 nm; methylene blue at 980 nm) that fall outside the conventional absorption maxima of the respective photosensitizers; because this is a central technological issue for whether these interventions should be interpreted as genuine photosensitizer-mediated PDT, these three combinations require verification against the original articles before the general statement that wavelengths “matched” the photosensitizers can be confirmed for the full dataset. Several studies also employed dual-light systems that combined 405-nm antibacterial blue light with 810-nm near-infrared irradiation [30,31,35].

Irradiation parameters also differed substantially in power output, irradiance, exposure time, and operating mode. Conventional chairside protocols generally involved irradiation periods ranging from several seconds to several minutes per site or surface, whereas dual-light protocols used 10-min treatment sessions and were frequently administered repeatedly at home over extended treatment periods. Most studies did not explicitly report the operating mode; continuous irradiation was specified in selected protocols, and pulsed irradiation was used in the 980-nm laser protocol. As summarized in Table 5, radiant exposure (J/cm^2^) could be calculated or was explicitly reported for most protocols, but not for all (e.g., Mongardini et al. [27], Vakaki et al. [30], and Malgikar et al. [37], for which irradiance or irradiated spot area was not reported), limiting comparison of delivered photodynamic dose across all studies since power output alone is not an adequate dose descriptor.

Overall, the considerable heterogeneity in light sources, wavelengths, irradiation doses, exposure times, and treatment schedules limits direct comparison between studies and may contribute to variability in the observed clinical and microbiological effects.

### 3.5. Photosensitizers and Adjunctive Enhancement Strategies

A broad range of photosensitizers was evaluated across the included studies (Table 5). Methylene blue was the most frequently investigated photosensitizer, followed by indocyanine green (ICG), while toluidine blue, riboflavin, and curcumin were used less frequently. Photosensitizer concentrations varied considerably between protocols and were reported in inconsistent units (μM, μg/mL, %, and g/L); as shown in Table 5, converting these to an approximate common molar-equivalent scale highlights several concentrations that are markedly higher than the remainder of the dataset and warrant verification against the original reports. Corresponding light sources and irradiation parameters also varied. Methylene blue was predominantly activated using red or near-infrared diode lasers, ICG was used with dual-light systems combining 405- and 810-nm irradiation, and toluidine blue was activated using red LED devices at approximately 628–630 nm.

The strategies used to enhance aPDT efficacy were diverse and reflected several distinct mechanistic approaches (Table 6). Physicochemical enhancement involved sodium dodecyl sulfate (SDS), a surfactant and permeabilizing agent that facilitated photosensitizer-biofilm interactions; together with nanocarrier- and oxygen-based approaches, this was classified as a direct enhancer acting on the photodynamic process itself (Section 2.3). Nanocarrier-based approaches employed polymeric nanoparticles, sometimes incorporated into *Aloe vera* gel, to improve photosensitizer retention, delivery, and interaction with target tissues. An oxygen-releasing formulation was used to increase local oxygen availability and potentially enhance ROS generation. Biological enhancement strategies included local and systemic probiotic supplementation and oral vitamin D3 supplementation, targeting microbiome modulation or host inflammatory and healing responses, respectively; together with adjunctive LLLT, these were classified as complementary adjuncts to an aPDT-containing protocol rather than direct modifiers of the photodynamic reaction. Light-based enhancement approaches included dual-light irradiation combining antibacterial blue light with near-infrared ICG-mediated aPDT, as well as adjunctive low-level laser therapy intended to provide photobiomodulatory effects. Collectively, these approaches targeted different potential limitations of conventional aPDT, including restricted photosensitizer penetration and retention, limited oxygen availability, biofilm tolerance, and host-related factors. The diversity of enhancement strategies further emphasizes the methodological heterogeneity of the included trials. It suggests that aPDT optimization has been approached through both direct modification of the photodynamic process and complementary modulation of the local biological environment.

## 4. Discussion

This systematic review demonstrates that attempts to enhance or complement aPDT in oral biofilm-associated conditions have evolved beyond modification of the conventional photosensitizer-light interaction and now encompass a broad range of physicochemical, delivery-based, light-based, biological, and host-modulating approaches. The available evidence does not support a uniformly beneficial effect of adjunctive strategies; rather, their effectiveness appears to depend on the mechanism of enhancement, the characteristics of the aPDT system, and the clinical context in which it is applied. Importantly, the heterogeneity of the investigated approaches suggests that aPDT enhancement should not be considered a single therapeutic concept, but rather a spectrum of strategies targeting different limitations of conventional photodynamic therapy. As detailed in Section 3.1, the eligible randomized evidence was also confined to bacterial biofilm-associated conditions; no eligible trial addressed an oral fungal infection, and the discussion below should be read with this restriction in mind.

### 4.1. Principal Findings and Interpretation of the Evidence

Given the mechanistic diversity of the investigated adjunctive strategies, their effects should be interpreted in relation to the specific component of the aPDT-based therapeutic process they are intended to modify or complement. Accordingly, the following discussion considers physicochemical enhancement of the photodynamic process; delivery-based and microenvironmental approaches; light-based and photobiomodulatory strategies; and biological and host-modulating adjuncts.

#### 4.1.1. Physicochemical Enhancement of the Photodynamic Process

Several chemical strategies have been proposed to enhance the efficacy of antimicrobial photodynamic therapy (aPDT), including inorganic salts (e.g., potassium iodide, potassium bromide, and thiocyanate), surfactants, and chelating agents designed to improve reactive oxygen species generation, photosensitizer stability, or microbial uptake. Among these approaches, surfactant-assisted formulations were the only chemical enhancement strategy evaluated in the randomized clinical trials included in the present systematic review and therefore constitute the primary focus of the following discussion [10,38].

Among the various surfactants investigated as chemical enhancers of antimicrobial photodynamic therapy, sodium dodecyl sulfate (SDS) is the most extensively studied. The primary role of surfactants is to facilitate the interaction between photosensitizers and microbial cells by improving photosensitizer dispersion, preventing molecular aggregation, and increasing permeability across bacterial membranes. These effects are particularly relevant for hydrophilic photosensitizers, whose intracellular accumulation is often hindered by the hydrophobic structure of bacterial cell envelopes, especially in Gram-negative microorganisms [39].

Several in vitro studies support these mechanistic observations. Methylene blue formulated with SDS demonstrated consistently greater antimicrobial activity against *Candida albicans* than methylene blue dissolved in water across a wide range of irradiation parameters. The improved efficacy was attributed to reduced methylene blue aggregation (metachromasy), thereby preserving its photodynamic activity even under conditions where body fluids might otherwise impair photosensitizer performance. However, excessive irradiation should be avoided, as prolonged light exposure may induce photobleaching of methylene blue and reduce treatment efficiency [40]. Importantly, the potentiating effect of SDS appears to be species-dependent rather than universal. While SDS-mediated antimicrobial photodynamic therapy produced complete elimination of *Porphyromonas gingivalis* after irradiation, largely owing to the intrinsic membrane-disrupting activity of the surfactant, no enhancement was observed against Aggregatibacter actinomycetemcomitans, for which conventional methylene blue-mediated aPDT in aqueous solution achieved greater bacterial reduction [41].

Surfactants as adjuncts were evaluated in four randomized clinical trials involving periodontal and orthodontic populations [24,25,29,32].

Overall, the available evidence suggests that the effectiveness of surfactant-enhanced aPDT depends not only on the presence of the surfactant itself but also on the physicochemical properties of the photosensitizer with which it is combined. The most convincing results were obtained in studies employing methylene blue and curcumin. Incorporating methylene blue into an SDS-based vehicle improved its antimicrobial performance under prolonged irradiation. Alvarenga et al. observed a significant 1.6-log reduction in bacterial survival after five minutes of irradiation with the SDS-containing formulation; as noted in Section 3.3, however, this formulation comparison was not itself randomized (randomization was applied only to irradiation time within each sequential cohort), so this finding is best regarded as supportive rather than confirmatory. In contrast, aqueous methylene blue produced no significant antimicrobial effect under the same irradiation conditions. Spectroscopic findings further suggested that SDS reduced methylene blue aggregation, with the SDS-containing formulation showing a single absorption peak corresponding to the monomeric form. These findings support the concept that surfactant-mediated enhancement may involve not only improved photosensitizer–microbial interactions but also favorable modifications of the photosensitizer’s photochemical properties [24].

Similarly, Panhóca et al. observed greater numerical reduction in salivary *S. mutans* with SDS-containing curcumin-mediated aPDT than with curcumin-mediated aPDT alone in orthodontic patients. The antimicrobial effect of the SDS-containing protocol was comparable with chlorhexidine, with no significant difference in bacterial survival between these interventions [29]. In contrast, two more recent randomized clinical trials, both sampling directly at the treated site rather than in saliva, failed to demonstrate additional clinical benefits from SDS supplementation. Kassa et al. did not demonstrate a statistically significant additional antimicrobial effect of SDS when added to methylene blue-mediated aPDT. Although the SDS-containing aPDT group showed the lowest bacterial survival fraction and an apparent reduction in colony growth, post hoc comparisons did not identify significant differences among individual treatment groups [25]. Likewise, Sudhakar et al. found no consistent enhancement of riboflavin-mediated aPDT following the addition of SDS. Riboflavin-mediated aPDT alone produced a more pronounced short-term reduction in *S. mutans*. In contrast, no significant differences between riboflavin-mediated aPDT with and without SDS were observed for *L. acidophilus*. Confocal microscopy further demonstrated the lowest residual biofilm viability with riboflavin-mediated aPDT alone and the highest with the riboflavin-SDS formulation [32]. These findings suggest that surfactant-mediated enhancement is not universally applicable to all photosensitizers.

Taken together, these apparently conflicting findings indicate that surfactant-mediated enhancement is highly formulation- and protocol-dependent. The favorable effects observed with SDS in some methylene blue- and curcumin-based protocols were not consistently reproduced across studies, whereas adding SDS to riboflavin-mediated aPDT provided no clear additional antimicrobial benefit. Differences in photosensitizer chemistry, aggregation behavior, surfactant-photosensitizer interactions, and irradiation conditions may contribute to this variability. Consequently, SDS should not be regarded as a universal enhancer of antimicrobial photodynamic therapy, but rather as a formulation-specific modifier whose effectiveness depends on the photosensitizer and treatment protocol.

#### 4.1.2. Delivery-Based and Microenvironmental Enhancement

##### Nanocarriers

Nanocarriers represent a widely investigated approach for improving photosensitizer delivery in aPDT. A variety of nanosystems, including metallic, silica, lipid-based, polymeric, and chitosan-based carriers, have been explored for the treatment of oral biofilm-associated infections [10,42]. Their potential advantages include improved photosensitizer solubility and stability, increased local retention and tissue penetration, protection from degradation, and the possibility of controlled or targeted delivery [42,43,44].

Beyond their role as delivery vehicles, nanocarriers may enhance interactions between photosensitizers and microbial cells. Experimental studies have demonstrated that positively charged photosensitizer-containing nanoparticles can promote bacterial surface association and increase local photodynamic activity [45]. Chitosan-based systems have similarly demonstrated antimicrobial activity against periodontal, peri-implant, and cariogenic microorganisms, including *A. actinomycetemcomitans* and *S. mutans* [46,47,48]. However, the persistence of mature biofilm resistance despite nanoparticle-mediated delivery indicates that improved photosensitizer delivery does not necessarily overcome all biological barriers associated with established biofilms [49,50].

Supporting clinical observations have also been reported with chitosan-based nanocarriers outside the randomized trials included in the present review. Hayashi et al. observed an immediate reduction in viable subgingival bacteria following chitosan-coated indocyanine green-mediated aPDT. However, the antimicrobial effect was largely transient, suggesting that improved local photosensitizer delivery may not necessarily provide sustained microbial suppression [51]. Similarly, Rose Bengal-functionalized chitosan nanoparticles reduced viable bacterial counts in deep carious lesions. However, chlorhexidine remained more effective and the enhanced bacterial reduction did not translate into improved short-term restorative outcomes [52]. These observations support the potential of nanocarrier-assisted photosensitizer delivery while also illustrating that greater immediate antimicrobial activity may not necessarily translate into sustained clinical benefit.

Within the randomized trials included in the present review, Qamar et al. and Elsadek et al. investigated photosensitizer-loaded polymeric nanoparticles incorporated into an *Aloe vera* gel matrix. Both studies demonstrated greater improvements in selected clinical parameters with the nanocarrier-based formulations than with conventional aPDT, particularly in probing depth and attachment- or bone-related outcomes. However, these clinical advantages were not consistently accompanied by superior microbiological effects, with conventional aPDT producing greater reductions in selected periodontal pathogens in both studies [26,33].

Importantly, the formulations investigated in these trials combined nanoparticle-mediated photosensitizer delivery with a biologically active *Aloe vera* vehicle. *Aloe vera* has been reported to have anti-inflammatory, antimicrobial, antioxidant, and wound-healing properties; therefore, it cannot necessarily be regarded as an inert carrier [53]. Previous clinical studies evaluating locally applied *Aloe vera*, independent of aPDT, have reported improvements in periodontal parameters and reductions in inflammatory mediators when used adjunctively with conventional periodontal treatment [54,55]. Its incorporation into hydrogel systems has also been explored as a means of combining local delivery with intrinsic biological activity [56].

Consequently, the favorable clinical effects observed by Qamar et al. and Elsadek et al. cannot be attributed exclusively to nanoparticle-mediated enhancement of photosensitizer delivery. The *Aloe vera* matrix may itself have contributed to the therapeutic response while potentially supporting local retention of the nanoparticle formulation. Because neither trial included comparator groups that would allow the effects of the nanoparticles and the *Aloe vera* vehicle to be evaluated separately, the relative contribution of each component and the possibility of synergistic interactions remain uncertain [26,33]. This confounding between nanocarrier and bioactive-vehicle effects is highlighted here with the same emphasis given to it in the Abstract, given its relevance to interpreting the clinical benefits reported for nanocarrier-based enhancement in Section 3.3.

Overall, the available evidence supports the use of nanocarriers primarily as delivery-modifying platforms rather than as universal amplifiers of antimicrobial efficacy. Their effects appear to depend on carrier composition, photosensitizer characteristics, biofilm maturity, treatment protocol, and the presence of additional bioactive components. Multifunctional formulations that combine nanocarrier-mediated delivery with bioactive vehicles such as *Aloe vera* may provide complementary therapeutic effects; however, their complexity also makes it difficult to determine which component is primarily responsible for the observed clinical benefit.

##### Oxygen-Enhancing Strategies

One of the fundamental limitations of antimicrobial photodynamic therapy is its dependence on molecular oxygen. Type II photochemical reactions directly require molecular oxygen to generate singlet oxygen, whereas Type I reactions initially proceed through electron or hydrogen transfer and can subsequently generate oxygen-derived radicals (e.g., superoxide and hydroxyl radicals); the statement that both reaction types “require” oxygen is therefore an oversimplification, since Type I photochemistry can, at least initially, proceed independently of oxygen before oxygen-dependent radical propagation occurs. Both pathways nonetheless contribute to the reactive oxygen species (ROS) responsible for microbial cell destruction in aPDT [8,9,10,18,57]. Consequently, oxygen depletion during light irradiation may still progressively reduce overall ROS production and limit the antimicrobial efficacy of aPDT, particularly for photosensitizers that act predominantly through Type II photochemistry, and especially in mature biofilms and deep periodontal pockets with hypoxic microenvironments [58].

Recent research has therefore shifted from improving photosensitizer delivery alone toward overcoming local oxygen deficiency. Rather than increasing light dose or photosensitizer concentration—which may promote photobleaching or tissue overheating—novel oxygen-enhancing strategies aim to maintain sufficient oxygen availability throughout the photodynamic reaction. Examples include oxygen-releasing gels, hydrogen peroxide-assisted protocols, oxygen-generating nanoparticles (e.g., manganese dioxide-based systems), perfluorocarbon oxygen carriers, and catalytic nanozymes that continuously produce oxygen from endogenous hydrogen peroxide, thereby sustaining ROS generation during irradiation [59,60,61].

Growing experimental evidence supports the rationale for these approaches. Multifunctional manganese dioxide-coated nanoplatforms capable of catalytically generating oxygen from endogenous hydrogen peroxide have been shown to alleviate local hypoxia, increase singlet oxygen production, and enhance bacterial killing by approximately one additional log compared with conventional aPDT. Beyond improving bacterial eradication, these oxygen-self-sufficient systems shifted multispecies periodontal biofilms toward a less pathogenic composition by reducing the abundance of anaerobic species such as *P. gingivalis* and *F. nucleatum*, while simultaneously decreasing local pro-inflammatory cytokine expression [62].

Hydrogen peroxide has likewise emerged as a simple and clinically attractive oxygen-enhancing adjunct. In endodontic biofilms, pre-treatment with hydrogen peroxide before methylene blue-mediated aPDT resulted in substantially greater bacterial inactivation than conventional protocols, thereby increasing the reduction in *E. faecalis* biofilms in vitro [63]. Similar findings have been reported for methicillin-resistant *Staphylococcus aureus*, in which hydrogen peroxide preoxidation synergized with curcumin-mediated aPDT, ultimately achieving complete bacterial eradication under optimized conditions [64]. Collectively, these findings suggest that improving oxygen availability may enhance aPDT not only by increasing ROS generation but also by overcoming hypoxia-associated resistance characteristic of mature biofilms.

In the only randomized controlled trial included in the present review, an active oxygen-releasing gel was evaluated alone and in combination with methylene blue-mediated aPDT in patients with poorly controlled type 2 diabetes and residual periodontal pockets. As noted in Section 3.3, this trial did not include a conventional aPDT-only comparator arm, so its findings should be interpreted as evidence for the combined oxygen-releasing gel + aPDT protocol rather than as isolated evidence for oxygen enhancement of aPDT. Favorable within-group changes in periodontal parameters were observed during follow-up, and the combined protocol was associated with a transient reduction in TNF-α levels at 90 days. However, no statistically significant between-group differences were demonstrated for the evaluated clinical periodontal parameters, and the additional clinical contribution of oxygen enhancement to aPDT therefore remains uncertain [28]. Overall, oxygen modulation represents a biologically plausible strategy to enhance aPDT, supported by experimental evidence showing improved ROS generation and antimicrobial activity under oxygen-enriched conditions. Nevertheless, this mechanistic rationale has not yet translated into clearly demonstrated additional clinical efficacy in an appropriately controlled randomized trial. Oxygen-enhancing approaches should therefore be regarded as mechanistically promising but clinically insufficiently validated. Further randomized trials, including a conventional aPDT-only comparator arm, are needed to determine whether optimization of oxygen delivery, treatment timing, and photodynamic parameters can translate the theoretical advantages of oxygen modulation into clinically meaningful benefits.

#### 4.1.3. Light-Based and Photobiomodulatory Strategies

##### Dual-Light Photodynamic Therapy

In addition to optimizing photosensitizers and adjunctive compounds, increasing attention has been paid to modifying the irradiation strategy itself. Conventional antimicrobial photodynamic therapy typically relies on a single excitation wavelength selected based on the photosensitizer’s absorption spectrum. In contrast, dual-light approaches combine different wavelengths within the same treatment protocol to engage complementary light-mediated antimicrobial mechanisms [65,66,67].

The dual-light approach evaluated in the included studies combines near-infrared irradiation at 810 nm with antibacterial blue light at 405 nm. The near-infrared component activates exogenous indocyanine green (ICG) associated with the biofilm, producing photodynamic and, potentially, photothermal antimicrobial effects, whereas 405-nm blue light is absorbed by endogenous bacterial chromophores, including porphyrins and flavins, thereby generating additional intracellular oxidative stress independently of the externally applied photosensitizer [65,66,67,68]. Because this protocol combines exogenous ICG-mediated photodynamic activation, endogenous chromophore-mediated blue-light photo-oxidation, and a possible photothermal component within a single treatment session, it should be regarded as a hybrid light-mediated antimicrobial system rather than a simple modification of conventional single-wavelength, single-mechanism aPDT; this distinction limits direct comparison of the dual-light findings discussed below with those obtained using classical photosensitizer-mediated aPDT elsewhere in this review. Thus, the principal rationale of dual-light therapy is the simultaneous combination of ICG-mediated photodynamic activity with a photosensitizer-independent antibacterial blue-light effect, delivered as an integrated hybrid protocol.

This complementary mechanism may offer several advantages over single-wavelength irradiation. Simultaneous targeting of exogenous and endogenous photosensitizing pathways may increase oxidative stress within microbial cells and broaden the antimicrobial effect across different components of the biofilm. Blue light may be particularly effective against microorganisms with endogenous photosensitive chromophores, whereas near-infrared light offers greater tissue penetration and more efficient ICG activation. Together, these properties may contribute to improved antimicrobial activity within structurally complex oral biofilms [18,65].

Repeated application may represent another relevant feature of the dual-light approach. Stable, heritable antimicrobial resistance to aPDT is generally considered unlikely because ROS affect multiple cellular targets simultaneously; however, this is mechanistically distinct from reversible adaptation or tolerance, in which repeated sublethal exposure transiently reduces a biofilm’s susceptibility to a given antimicrobial modality without a fixed genetic change. Experimental studies suggest that repeated exposure to individual light-based antimicrobial modalities may induce such reversible tolerance in mature biofilms. In vitro, repeated administration of either conventional aPDT or antibacterial blue light alone resulted in a progressive reduction in antibacterial efficacy against mature biofilms of *S. mutans* and *S. aureus*, consistent with adaptive tolerance rather than classical resistance. In contrast, simultaneous dual-light treatment maintained antimicrobial activity during repeated exposure, suggesting a lower propensity for this biofilm adaptation response; these experimental findings reinforce the need for precise terminology distinguishing reversible tolerance from stable antimicrobial resistance when describing aPDT’s resistance profile [66,67].

In addition to these direct antimicrobial mechanisms, a potential photobiomodulatory contribution of the near-infrared component should be considered. Near-infrared light in this wavelength range may interact with host tissues independently of photosensitizer activation, influencing cellular processes related to inflammation, tissue repair, and wound healing [68]. This potential effect is conceptually distinct from the principal dual-light antibacterial mechanism described above. Importantly, however, the available clinical trials evaluated the complete hybrid dual-light protocol and were not designed to isolate the contribution of photobiomodulation from the simultaneous photodynamic, potentially photothermal, and antibacterial effects of blue light [69]. The clinical relevance of this additional mechanism therefore remains uncertain.

Three randomized controlled trials included in the present review evaluated repeated home application of dual-light aPDT in periodontal or peri-implant conditions [30,31,35]. Adjunctive dual-light aPDT combined with non-surgical periodontal treatment resulted in a greater reduction in bleeding on probing (BOP), a higher proportion of patients achieving the BOP < 10% target, and greater reductions in deep periodontal pockets after three months compared with non-surgical periodontal treatment alone [31]. Favorable effects were also observed in peri-implant disease, where four weeks of home-applied dual-light aPDT was associated with a marked reduction in BOP and a decrease in aMMP-8 levels compared with enhanced self-care alone. Although reductions in the deepest peri-implant pockets occurred in some treated patients, between-group differences in probing depth were not statistically significant [35]. In contrast, in smokers with Stage III-IV periodontitis, significant improvements in BOP, probing depth, and CAL occurred over time in both treatment groups. Still, no statistically significant between-group differences in the principal periodontal outcomes were demonstrated at later follow-up assessments, indicating no clear additional clinical benefit of dual-light aPDT over non-surgical periodontal treatment in this population [30].

Taken together, the available randomized evidence provides preliminary but inconsistent support for repeated home-use dual-light aPDT as an adjunct in periodontal and peri-implant therapy. The most favorable findings were for inflammatory outcomes, particularly BOP, whereas additional improvements in probing depth and CAL were less consistently observed. Differences in disease characteristics, smoking status, baseline plaque control, treatment duration, and adherence to home use may partly contribute to the variability between studies. Although photobiomodulation may theoretically contribute to some tissue-level effects, its independent contribution cannot be determined from the available trials because of the hybrid, multi-mechanism nature of the dual-light protocol. Thus, despite a strong mechanistic rationale and some favorable clinical signals, the limited number of eligible randomized trials and the inconsistency of their findings preclude firm conclusions regarding the incremental clinical benefit of dual-light aPDT over conventional periodontal therapy.

##### Adjunctive Low-Level Laser Therapy

Low-level laser therapy (LLLT) represents a light-based adjunctive approach that differs mechanistically from the direct photosensitizer-mediated antimicrobial action of aPDT. Its therapeutic rationale is primarily associated with photobiomodulation, with reported effects on inflammation, cell proliferation, tissue repair, and wound healing. Although direct antibacterial effects have also been reported, their magnitude appears to depend on the microbial species and irradiation conditions, suggesting that the principal role of LLLT in combined periodontal protocols may be to complement antimicrobial treatment by modulating the host tissue response [70,71].

Importantly, photobiomodulatory effects are strongly dependent on irradiation parameters. In vitro studies have demonstrated wavelength-dependent cellular responses, with visible red wavelengths above 665 nm stimulating fibroblast and endothelial cell proliferation, whereas 810-nm irradiation inhibited fibroblast proliferation under the investigated conditions [72]. Thus, photobiomodulatory effects cannot be inferred from wavelength alone and may vary according to irradiation parameters and the biological target.

In the only randomized controlled trial included in the present review, Malgikar et al. evaluated LLLT as an adjunct to SRP and methylene blue-mediated aPDT in patients with chronic periodontitis. Although all treatment approaches improved periodontal parameters, the combined SRP + aPDT + LLLT protocol produced greater reductions in probing depth and clinical attachment gain than SRP + aPDT at the 6-month follow-up, with generally more pronounced improvements in other periodontal indices [37]. These findings suggest that LLLT may provide a photobiomodulatory complement to the antimicrobial effects of aPDT; however, evidence is currently limited to a single randomized trial. LLLT should therefore be regarded as a promising complementary light-based strategy rather than an established enhancer of the direct antimicrobial action of aPDT.

#### 4.1.4. Biological and Host-Modulating Adjuncts

##### Probiotic Adjuncts

Biological adjuncts represent a distinct approach to complementing aPDT by modifying the local microbial ecosystem rather than directly altering photosensitizer delivery or photochemical reactions. Probiotic and postbiotic strategies may interfere with pathogen growth, adhesion, biofilm organization, and recolonization, potentially complementing the rapid oxidative antimicrobial activity of aPDT. Experimental studies support this concept, demonstrating enhanced photodynamic inactivation and inhibition of biofilm development when aPDT is combined with probiotic-derived metabolites or selected probiotic microorganisms [73,74].

Clinical evidence, however, remains limited and inconsistent and is restricted to what the included trials actually measured—principally reductions in selected periodontal pathogens and clinical inflammatory indices—rather than a comprehensive assessment of the oral microbiome; reductions in one or two targeted pathogens should not be equated with restoration or broader modulation of the overall oral microbial community, which was not assessed in either trial. In experimentally induced peri-implant mucositis, adjunctive administration of *L. plantarum* and *L. brevis* provided no significant additional clinical benefit over professionally administered plaque removal and aPDT, with comparable reductions in plaque accumulation and bleeding following probiotic and placebo treatment [27]. In periodontitis, more favorable effects were observed when probiotics were added to subgingival debridement and light-activated disinfection, resulting in a significantly greater reduction in BoP than the other treatment approaches at both 3 and 6 months. Gingival inflammation was also lower than with light-activated disinfection without probiotics at 6 months, and greater reductions in the two specifically measured pathogens, *P. gingivalis* and *T. forsythia*, were observed compared with the other treatment groups at that time point; probing depth and clinical attachment level did not differ significantly between groups, and no broader microbiome-level analysis was performed [34].

Additional clinical evidence outside the eligible randomized trials is broadly consistent with the absence of a uniform adjunctive effect. A single application of probiotics combined with PDT and scaling and root debridement did not significantly improve probing pocket depth or clinical attachment level compared with mechanical treatment alone at three months [75]. Although this study was not included in the present review, its findings provide additional clinical context and suggest that any benefit of probiotic supplementation may depend on factors such as treatment frequency, duration of probiotic exposure, and the overall intervention protocol.

Taken together, the available evidence suggests that probiotic supplementation may preferentially influence inflammatory outcomes and the abundance of specific, individually measured pathogens rather than consistently improving probing depth or clinical attachment gain, and current trials do not provide direct evidence of broader oral microbiome restoration or modulation. The contrasting findings across clinical studies may partly reflect differences in disease model, probiotic administration, treatment duration, and aPDT/LAD protocols. Thus, biological modulation remains a promising complementary strategy, but the small and heterogeneous clinical evidence base does not currently support a consistent additional benefit of probiotics when combined with aPDT, nor conclusions about microbiome-level effects beyond the specific pathogens measured.

##### Host-Modulating Adjuncts: Vitamin D

Vitamin D represents a host-modulating adjunct with potential relevance to periodontal therapy through its role in bone metabolism, immune regulation, and inflammatory control. Adequate vitamin D status has been associated with periodontal health, while supplementation has been proposed to support periodontal tissues by promoting bone homeostasis and limiting alveolar bone resorption [76]. Vitamin D may also contribute to antimicrobial host defense by regulating endogenous antimicrobial peptides, including cathelicidins and defensins, thereby providing a potential link between immune modulation and microbial control [77].

In the randomized trial included in the present review, oral vitamin D supplementation added to an otherwise identical NSPT and methylene blue-mediated PDT protocol resulted in greater short-term periodontal improvement. Clinical attachment level improved significantly in the vitamin D group, with a significant between-group difference emerging at 6 weeks, while plaque and gingival indices showed greater reductions than with NSPT and PDT alone [36].

These findings suggest that vitamin D may complement aPDT-containing periodontal treatment primarily through host-modulating and periodontal-supportive effects rather than by directly enhancing the photodynamic antimicrobial reaction. However, as the available evidence is limited to a single randomized trial and no direct mechanistic or microbiological evidence of enhanced photodynamic activity was demonstrated, vitamin D should currently be regarded as a promising systemic adjunct rather than a confirmed enhancer of aPDT.

### 4.2. Limitations of the Evidence

The current clinical evidence regarding adjunctive strategies to enhance or complement antimicrobial photodynamic therapy remains limited due to substantial methodological and clinical heterogeneity. Although the present review was restricted to randomized controlled trials, the included studies investigated mechanistically distinct approaches, encompassing physicochemical, delivery-based and microenvironmental, light-based and photobiomodulatory, and biological and host-modulating strategies. Considerable variability was also observed within these categories with respect to photosensitizer type and concentration, light source, irradiation wavelength and dose, exposure time, treatment frequency, adjunct formulation, and comparator intervention. This heterogeneity limits direct comparison across studies and makes it difficult to determine whether differences in treatment effects reflect the adjunctive strategy itself or the characteristics of the underlying aPDT protocol.

The clinical indications represented in the available randomized evidence were also heterogeneous, including periodontal diseases of varying severity, peri-implant conditions, and orthodontic biofilm control; notably, despite the review question encompassing both bacterial and fungal oral biofilm-associated disease, no eligible randomized trial addressing a fungal condition (e.g., oral candidiasis) was identified, so the conclusions of this review apply to bacterial biofilm-associated disease only and should not be extrapolated to oral candidiasis or other fungal biofilms. These settings also differ in microbial composition, biofilm organization, tissue environment, therapeutic objectives, and outcome assessment. Consequently, findings obtained for a particular enhancement strategy in one clinical context cannot necessarily be extrapolated to another, and the available evidence does not permit identification of a universally superior adjunctive approach.

The multifunctional nature of some interventions further complicates interpretation. This is particularly relevant to the nanocarrier-based formulations evaluated in the included trials, in which photosensitizer-loaded polymeric nanoparticles were incorporated into an *Aloe vera* gel matrix. Because *Aloe vera* itself possesses potentially relevant anti-inflammatory, antimicrobial, and wound-healing properties, the observed clinical effects cannot be attributed exclusively to nanoparticle-mediated photosensitizer delivery. The absence of comparator groups isolating the individual formulation components further prevents determination of their relative contributions or possible synergistic effects.

Another limitation is the generally short follow-up period. Most studies evaluated outcomes from the immediate post-treatment period through 6 months, providing limited information on the persistence of clinical or microbiological benefits. In addition, microbiological and immunological outcomes were reported and assessed inconsistently, with different analytical methods (culture/CFU, species-specific molecular quantification, and CLSM-based viability imaging) used across trials and not always fully distinguished in the primary reports, further reducing comparability. Although treatment-related adverse events were uncommon and the evaluated protocols appeared generally well-tolerated where safety was reported (Table 4), safety outcomes were not systematically reported across all included trials, and the available studies were not primarily designed or adequately powered to establish long-term safety, particularly for SDS-containing formulations, nanoparticle-based delivery systems, and repeated home-use light protocols.

Taken together, the available randomized evidence supports the potential of several adjunctive approaches but does not establish a consistently effective enhancement strategy across different aPDT systems and clinical indications. The observed effects should therefore be interpreted as strategy-, formulation-, and context-dependent rather than as evidence for a general benefit of “enhanced aPDT” as a single therapeutic concept.

### 4.3. Limitations of the Review Process

Several limitations of the present systematic review should also be acknowledged. First, despite restricting eligibility to randomized controlled trials, substantial clinical and methodological heterogeneity precluded quantitative synthesis; the predefined considerations underlying this decision are detailed in Section 2.5. Differences in clinical indications, photosensitizers, irradiation parameters, adjunctive interventions, comparator groups, follow-up periods, and outcome definitions precluded meaningful meta-analysis and limited direct comparison of effect magnitudes across enhancement strategies. Related to this, the review relied on a narrative synthesis without a formal certainty-of-evidence framework; incorporating a structured approach such as GRADE, adapted as appropriate for narrative syntheses of heterogeneous randomized evidence, could enhance the transparency and clinical translatability of the confidence assigned to each enhancement category in future updates of this review.

Second, the clinical evidence base remains small for most individual strategies. Some approaches, including oxygen-enhancing formulations, adjunctive low-level laser therapy, and vitamin D supplementation, were represented by a single randomized trial. At the same time, other strategies were evaluated in only a small number of studies. Consequently, conclusions regarding individual adjuncts should be considered preliminary, particularly where findings have not been independently replicated across different populations and treatment protocols.

Third, only English-language publications were included, introducing the possibility of language bias. Gray literature, conference abstracts, and unpublished studies were also excluded, potentially increasing the risk of publication bias. An additional limitation arises from the rapid development of technologies aimed at improving aPDT. Several mechanistically promising approaches—including inorganic salts, oxygen-generating nanoplatforms, advanced nanomaterials, and novel photosensitizer delivery systems—remain supported predominantly by experimental or preclinical evidence and therefore cannot contribute directly to clinical synthesis. Accordingly, this review reflects the current randomized clinical evidence rather than the full range of enhancement strategies under investigation.

Finally, although the methodological quality of several included trials was acceptable, the risk-of-bias assessment identified concerns in several studies, including incomplete reporting of allocation procedures, irradiation parameters, and microbiological outcomes; the RoB 2 assessments themselves are outcome- and time-point-specific, as detailed in Table 3 and Section 2.6. These limitations reduce reproducibility and should be considered when interpreting the confidence that can be placed in the findings, which—in the absence of a formal certainty assessment—is limited specifically by the identified risk of bias and by the clinical and methodological heterogeneity described above, rather than by an “overall certainty” in the GRADE sense.

### 4.4. Implications for Clinical Practice and Future Research

The findings of this review suggest that adjunctive strategies may improve selected clinical or microbiological outcomes when incorporated into aPDT-based treatment; however, their effects are not uniform and appear to depend on the mechanism of the adjunct, the photosensitizer and formulation used, irradiation parameters, treatment frequency, and clinical indication. Current evidence therefore does not support the routine use of a single enhancement strategy across all aPDT applications. Given the small evidence base for most individual strategies and the absence of a formal certainty-of-evidence assessment (Section 4.3), the interventions discussed below are best regarded as potentially useful, investigational adjuncts rather than established options that clinicians may routinely select among.

From a clinical perspective, the available findings are best interpreted in light of the therapeutic limitation targeted by each strategy. Surfactant-assisted formulations may improve photosensitizer performance under specific conditions, but their effects appear to be strongly dependent on the photosensitizer chemistry and irradiation protocol. Nanocarrier-based formulations may improve local delivery and have been associated with favorable changes in selected periodontal and peri-implant clinical outcomes; however, superior microbiological efficacy has not been consistently demonstrated, and the contribution of accompanying bioactive vehicles remains uncertain. Oxygen-enhancing approaches have a strong mechanistic rationale but currently lack convincing evidence of additional clinical efficacy from an appropriately controlled trial. Repeated dual-light protocols have shown favorable effects, particularly on inflammatory and plaque-related outcomes in some periodontal and peri-implant populations, whereas improvements in probing depth and clinical attachment have been less consistent. LLLT, probiotics, and vitamin D may provide complementary photobiomodulatory, pathogen-specific microbiological, or host-modulating effects, respectively, but the clinical evidence supporting these approaches remains limited to one or two trials each.

Importantly, none of these strategies should currently be regarded as a replacement for established mechanical biofilm disruption. Rather, they represent investigational adjunctive approaches that may warrant consideration, within appropriately designed clinical protocols and shared decision-making, to address specific limitations of aPDT or to complement its antimicrobial effects in particular clinical settings, pending further confirmatory evidence.

Future randomized clinical trials should prioritize both methodological standardization and mechanism-oriented study design. Standardized reporting of photosensitizer concentration, formulation, irradiation wavelength, irradiance and energy dose, exposure time, treatment frequency, and microbiological and clinical outcomes would improve comparability between studies. Where multifunctional formulations are evaluated, appropriate comparator groups should be included to distinguish the contribution of individual components, such as the nanocarrier, bioactive vehicle, or photosensitizer modification, and trials evaluating adjuncts added to an aPDT-containing protocol should include a conventional aPDT-only arm wherever feasible so that the independent contribution of the adjunct can be isolated. Further studies should also include adequately powered samples, longer follow-up periods, standardized and clearly specified microbiological methods (culture/CFU, species-specific molecular quantification, or viability imaging) and clinical outcomes, patient-reported outcomes, systematic safety assessment, and cost-effectiveness analyses. Direct comparisons between enhancement strategies may be informative where clinically and mechanistically appropriate; however, priority should also be given to replicating promising approaches using comparable protocols before attempting to draw conclusions about their relative superiority. Trials addressing fungal oral biofilm-associated conditions are also needed, since none met the eligibility criteria of the present review despite the original PICO question encompassing fungal disease.

Finally, a substantial translational gap remains between the diversity of enhancement mechanisms demonstrated experimentally and the comparatively limited randomized clinical evidence. Strategies such as oxygen-generating nanoplatforms, inorganic salts, advanced carrier systems, and other formulation modifiers have shown promising mechanistic effects but require rigorous clinical validation. Future research should therefore focus not only on developing increasingly complex enhancement systems, but also on determining which specific limitations of aPDT they address and whether these mechanistic advantages translate into clinically meaningful and durable benefits.

## 5. Conclusions

This systematic review indicates that enhancement of antimicrobial photodynamic therapy should not be considered a single therapeutic concept. Rather, the investigated adjunctive approaches target different components or limitations of aPDT-based treatment, including photosensitizer behavior and microbial interaction, local delivery and retention, oxygen availability, irradiation-related antimicrobial mechanisms, biofilm ecology, and host tissue responses. Their clinical effects should therefore be interpreted according to the specific mechanism and treatment context in which they are applied. The available randomized evidence was drawn overwhelmingly from periodontal, peri-implant, and orthodontic populations, and no eligible randomized trial addressed a fungal oral biofilm-associated condition; the conclusions below should therefore not be extrapolated to oral candidiasis or other fungal biofilms.

Across the available randomized evidence, no adjunctive strategy demonstrated consistent superiority across clinical indications and outcome domains. Surfactant-mediated enhancement was strongly dependent on the photosensitizer and treatment protocol. Nanocarrier-based formulations produced favorable effects on selected clinical outcomes, although superior microbiological effects did not consistently accompany these and could not be attributed exclusively to nanoparticle-mediated delivery given the confounding contribution of the co-administered *Aloe vera* vehicle. Oxygen-enhancing strategies remain mechanistically promising but clinically insufficiently validated and have not yet been tested against a conventional aPDT-only comparator. Repeated dual-light protocols—which function as hybrid, multi-mechanism light-based systems rather than simple modifications of conventional aPDT—showed favorable effects on selected inflammatory outcomes, particularly bleeding on probing, although additional improvements in probing depth and clinical attachment level were not consistently demonstrated. Biological, photobiomodulatory, and host-modulating adjuncts, including probiotics, LLLT, and vitamin D, may complement aPDT through mechanisms distinct from direct enhancement of the photodynamic antimicrobial reaction, but the available evidence remains limited to one or two trials per strategy and, for probiotics, to the specific pathogens measured rather than to broader oral microbiome modulation.

Overall, current evidence supports a strategy-specific and mechanism-oriented approach to aPDT optimization rather than the concept of a universally effective enhancer, and the interventions reviewed here are best regarded as investigational adjuncts pending further confirmatory trials rather than as established treatment options. The clinical evidence remains limited by heterogeneous protocols, relatively few randomized trials for individual strategies, the absence of a formal certainty-of-evidence assessment, and limited long-term follow-up. Future research should therefore prioritize standardized, adequately powered randomized trials—including trials in fungal oral biofilm-associated disease—longer observation periods, mechanistically informative comparator groups (including conventional aPDT-only arms where adjuncts are added to an aPDT-containing protocol), and the rational matching of adjunctive strategies to specific aPDT limitations, photosensitizers, and clinical indications. Such an approach will be necessary to determine whether promising mechanistic enhancements translate into reproducible and clinically meaningful benefits.

## Figures and Tables

**Table 1:** Search syntax used in the study.

Source	Search Term	Filters	Number of Results
PubMed	(“Photochemotherapy”[Mesh] OR “photodynamic therapy”[tiab] OR “antimicrobial photodynamic therapy”[tiab] OR “antimicrobial photodynamic treatment”[tiab] OR “antimicrobial photodynamic inactivation”[tiab] OR “antibacterial photodynamic therapy”[tiab] OR “photodynamic disinfection”[tiab] OR “photoactivated disinfection”[tiab] OR “photo-activated disinfection”[tiab] OR “photodynamic antimicrobial chemotherapy”[tiab] OR “light-activated disinfection”[tiab] OR “light activated disinfection”[tiab] OR APDT[tiab]) AND (“Periodontitis”[Mesh] OR “Periodontal Diseases”[Mesh] OR “Peri-Implantitis”[Mesh] OR “Gingivitis”[Mesh] OR “Dental Caries”[Mesh] OR “Endodontics”[Mesh] OR “Orthodontics”[Mesh] OR “Oral Candidiasis”[Mesh] OR periodont*[tiab] OR gingiv*[tiab] OR periimplant*[tiab] OR peri-implant*[tiab] OR endodont*[tiab] OR orthodont*[tiab] OR “root canal”[tiab] OR caries[tiab] OR carious[tiab] OR dentin*[tiab] OR denture*[tiab] OR candid*[tiab] OR “oral biofilm”[tiab] OR “dental biofilm”[tiab]) AND (randomized controlled trial[pt] OR controlled clinical trial[pt] OR randomised[tiab] OR randomized[tiab] OR randomly[tiab] OR placebo[tiab] OR trial[tiab]) NOT (animals[mh] NOT humans[mh])	Publication date: From 9 November 2015 to 9 November 2025 Language: English	562
Embase	(‘antimicrobial photodynamic therapy’/exp OR ‘photodynamic therapy’:ti,ab OR ‘antimicrobial photodynamic therapy’:ti,ab OR ‘antimicrobial photodynamic treatment’:ti,ab OR ‘antimicrobial photodynamic inactivation’:ti,ab OR ‘antibacterial photodynamic therapy’:ti,ab OR ‘photodynamic disinfection’:ti,ab OR ‘photoactivated disinfection’:ti,ab OR ‘photo-activated disinfection’:ti,ab OR ‘photodynamic antimicrobial chemotherapy’:ti,ab OR ‘light-activated disinfection’:ti,ab OR ‘light activated disinfection’:ti,ab OR apdt:ti,ab) AND (‘periodontitis’/exp OR ‘periodontal disease’/exp OR ‘periimplantitis’/exp OR ‘gingivitis’/exp OR ‘dental caries’/exp OR ‘endodontics’/exp OR ‘orthodontics’/exp OR ‘oral candidiasis’/exp OR periodont*:ti,ab OR gingiv*:ti,ab OR periimplant*:ti,ab OR ‘peri implant*’:ti,ab OR endodont*:ti,ab OR orthodont*:ti,ab OR ‘root canal’:ti,ab OR caries:ti,ab OR carious:ti,ab OR dentin*:ti,ab OR denture*:ti,ab OR candid*:ti,ab OR ‘oral biofilm’:ti,ab OR ‘dental biofilm’:ti,ab) AND (‘randomized controlled trial’/de OR ‘controlled clinical trial’/de OR random*:ti,ab OR randomly:ti,ab OR placebo*:ti,ab OR trial:ti) NOT (‘animal’/exp NOT ‘human’/exp)	Publication years: 2015–2025 Language: English	623
Scopus	(TITLE-ABS-KEY (“photodynamic therapy”) OR TITLE-ABS-KEY (“antimicrobial photodynamic therapy”) OR TITLE-ABS-KEY (“antimicrobial photodynamic treatment”) OR TITLE-ABS-KEY (“antimicrobial photodynamic inactivation”) OR TITLE-ABS-KEY (“antibacterial photodynamic therapy”) OR TITLE-ABS-KEY (“photodynamic disinfection”) OR TITLE-ABS-KEY (“photoactivated disinfection”) OR TITLE-ABS-KEY (“photo-activated disinfection”) OR TITLE-ABS-KEY (“photodynamic antimicrobial chemotherapy”) OR TITLE-ABS-KEY (“light-activated disinfection”) OR TITLE-ABS-KEY (“light activated disinfection”) OR TITLE-ABS-KEY (apdt)) AND (TITLE-ABS-KEY (periodont*) OR TITLE-ABS-KEY (gingiv*) OR TITLE-ABS-KEY (periimplant*) OR TITLE-ABS-KEY (peri-implant*) OR TITLE-ABS-KEY (endodont*) OR TITLE-ABS-KEY (orthodont*) OR TITLE-ABS-KEY (“root canal”) OR TITLE-ABS-KEY (caries) OR TITLE-ABS-KEY (carious) OR TITLE-ABS-KEY (dentin*) OR TITLE-ABS-KEY (denture*) OR TITLE-ABS-KEY (candid*) OR TITLE-ABS-KEY (“oral biofilm”) OR TITLE-ABS-KEY (“dental biofilm”)) AND (TITLE-ABS-KEY (random*) OR TITLE-ABS-KEY (randomly) OR TITLE-ABS-KEY (placebo*) OR TITLE (trial))	Years: 2015–2025 Language: English	726
Cochrane	(“photodynamic therapy” OR “antimicrobial photodynamic therapy” OR “antimicrobial photodynamic treatment” OR “antimicrobial photodynamic inactivation” OR “antibacterial photodynamic therapy” OR “photodynamic disinfection” OR “photoactivated disinfection” OR “photo-activated disinfection” OR “photodynamic antimicrobial chemotherapy” OR “light-activated disinfection” OR “light activated disinfection” OR apdt) AND (periodont* OR gingiv* OR periimplant* OR peri-implant* OR endodont* OR orthodont* OR “root canal” OR caries OR carious OR dentin* OR denture* OR candid* OR “oral biofilm” OR “dental biofilm”)	Years: 2015–2025 Language: English	664

**Table 2:** Eligibility criteria for study inclusion and exclusion.

Inclusion Criteria	Exclusion Criteria
1. Study design: Randomized controlled clinical trials (RCTs).	1. Study design: Non-randomized interventional studies, observational studies, case reports, case series, reviews, and other secondary research.
2. Population/condition: Studies involving patients with oral biofilm-related bacterial or fungal diseases, including periodontal, peri-implant, caries-related, or other eligible oral biofilm-associated conditions.	2. Population/condition: Studies not involving an oral biofilm-related bacterial or fungal disease, including conditions of primarily viral or non-microbial origin.
3. Intervention: Antimicrobial photodynamic therapy (aPDT) incorporating or combined with an adjunctive strategy intended to enhance the photodynamic process or complement the therapeutic antimicrobial effect of an aPDT-based intervention.	3. Intervention: Studies evaluating PDT for purposes other than antimicrobial treatment, conventional aPDT without an eligible enhancement or complementary strategy, or interventions in which aPDT did not constitute a defined component of the treatment protocol.
4. Enhancement strategy: Eligible strategies included physicochemical, delivery-based, light-based, biological, or host-modulating approaches acting either directly on components of the photodynamic process (direct enhancers) or as complementary adjuncts to an aPDT-containing treatment protocol.	4. Ineligible adjuncts: Conventional antimicrobial or supportive therapies administered solely as independent concomitant treatments, without a defined mechanistic or therapeutic rationale for enhancing or complementing the aPDT-based intervention.
5. Comparator: Studies were required to include an appropriate control or comparison condition permitting evaluation of the enhanced or complementary aPDT-based intervention, stratified as enhanced-vs-conventional aPDT or aPDT-containing-vs-standard-therapy comparisons.	5. Comparator: Studies lacking an appropriate control or comparison condition for evaluation of the enhanced or complementary aPDT-based intervention.
6. Outcomes: Studies reporting clinical and/or microbiological outcomes relevant to the antimicrobial treatment of the investigated oral biofilm-related disease.	6. Outcomes: Studies not reporting clinical or microbiological outcomes relevant to the antimicrobial therapeutic effect under investigation.
7. Publication characteristics: Full-text articles published in English between 9 November 2015 and 9 November 2025.	7. Publication characteristics: Articles published in languages other than English or outside the predefined publication period.
	8. Publication type: Conference abstracts without sufficient study data, protocols, and trial registry records were not considered independent eligible studies.

**Table 3:** Risk-of-bias assessment of the included studies (RoB 2).

Study	Outcome Assessed (Time Point)	Randomization Process	Deviations from Intended Interventions	Missing Outcome Data	Measurement of the Outcome	Selection of Reported Result	Overall
Alvarenga et al., 2019 [24]	Bacterial survival fraction (post-irradiation)	High	Low	Low	Low	Some concerns	High
Kassa et al., 2022 [25]	Bacterial survival/CFU (immediately post-treatment); PPD (30 days)	Low	Low	Some concerns	Low	Some concerns	Some concerns
Qamar et al., 2023 [26]	Peri-implant clinical/microbiological parameters (6 months)	Some concerns	Low	Low	Low	Some concerns	Some concerns
Mongardini et al., 2017 [27]	Modified PI and BOP-positive sites (per crossover phase)	Low	Low	Low	Low	Low	Low
Cláudio et al., 2024 [28]	Residual pockets PD ≥ 4 mm with BoP (90/180 days)	Low	Low	Low	Low	Low	Low
Panhóca et al., 2016 [29]	*S. mutans* salivary viability (post-aPDT)	Low	Low	Low	Low	Some concerns	Some concerns
Vakaki et al., 2025 [30]	BoP, PD, CAL (6 months)	Low	Some concerns	Some concerns	Some concerns	Low	Some concerns
Pakarinen et al., 2022 [31]	BoP, deep pocket reduction (3-month interim)	Low	Some concerns	Some concerns	Some concerns	Some concerns	Some concerns
Sudhakar et al., 2025 [32]	*S. mutans*/*L. acidophilus* counts (2-month follow-up)	Low	Low	Low	Low	Low	Low
Elsadek et al., 2023 [33]	PD, CAL, microbial counts (6 months)	Low	Low	Low	Some concerns	Some concerns	Some concerns
Patyna et al., 2021 [34]	BoP, PPD, CAL, pathogen counts (6 months)	Low	Low	Low	Low	Low	Low
Lähteenmäki et al., 2025 [35]	BoP, aMMP-8, PPD (4 weeks)	Some concerns	Some concerns	Low	Some concerns	Some concerns	Some concerns
El Refai et al., 2025 [36]	CAL, PI, GI (6 weeks)	Low	Some concerns	Low	Some concerns	Some concerns	Some concerns
Malgikar et al., 2016 [37]	PD, CAL (6 months)	Low	Low	Low	Some concerns	Some concerns	Some concerns

**Table 4:** Summary of the principal results, study details, and reported safety outcomes.

Author and Year	Study Groups	Outcomes	Safety/Adverse Events
Alvarenga et al., 2019 [24]	30 patients with chronic periodontitis were evaluated in two sequential, non-randomized cohorts. In the first cohort, 20 patients contributed 60 periodontal sites treated with aqueous MB, whereas 10 patients in the second cohort contributed 30 sites treated with MB in 0.25% SDS (MBS).	Aqueous MB-mediated aPDT did not significantly reduce bacterial survival at any irradiation time (1, 3, or 5 min). MBS-mediated aPDT produced a significant 1.6-log reduction in bacterial survival after 5 min of irradiation, whereas 1- and 3-min irradiation produced no significant reduction. Although MBS irradiation for 1 and 3 min did not significantly reduce CFU/mL, changes in bacterial colony growth patterns, including smaller colonies, were observed.	Not explicitly reported in the available data
Kassa et al., 2022 [25]	36 patients with periodontitis received basic periodontal treatment. They were randomized into four groups: aPDT with MB in SDS, aPDT with aqueous MB, MB in SDS without irradiation, and MB in aqueous solution without irradiation. Subgingival biofilm was sampled before and immediately after treatment, while PPD was reassessed after 30 days, enabling evaluation of the additional effect of SDS on MB-mediated aPDT.	aPDT with MB in the SDS group showed the lowest bacterial survival fraction and an apparent reduction in CFU/mL; however, although the overall between-group analysis was significant, post-hoc comparisons did not identify significant differences between individual groups. Aqueous MB-mediated aPDT did not produce an apparent reduction in bacterial colony counts, whereas the SDS-containing aPDT group showed reduced CFU/mL and smaller bacterial colonies after irradiation. No significant between-group differences in PPD changes were observed at the evaluated follow-up time points.	Not explicitly reported in the available data
Qamar et al., 2023 [26]	102 patients with chronic hyperglycemia and peri-implantitis were randomized into three groups (n = 34 each): MD combined with PGA/RF/AV, MD combined with conventional riboflavin-mediated aPDT, or MD alone. Clinical, radiographic, and microbiological outcomes were assessed at baseline, 3 months, and 6 months.	All three treatment groups showed reductions in peri-implant clinical and radiographic parameters at 3 and 6 months. The PGA/RF/AV + MD group demonstrated significantly greater reductions in plaque index, probing depth, and marginal bone level compared with the other treatment groups. A significant reduction in bleeding on probing was observed in the conventional PDT + MD group. *Porphyromonas gingivalis* counts were significantly reduced in the PDT + MD group at 3 and 6 months, whereas *Tannerella forsythia* counts were significantly reduced in both adjunctive treatment groups. The greatest overall reduction in microbial counts was observed in the conventional PDT + MD group at both 3 and 6 months.	Not explicitly reported in the available data
Mongardini et al., 2017 [27]	20 systemically healthy patients, each contributing one implant with experimentally induced peri-implant mucositis, received PAPR combined with aPDT. In a randomized cross-over design, participants received adjunctive probiotics (*Lactiplantibacillus plantarum* and *Levilactobacillus brevis*) or placebo, followed by a wash-out period and crossover to the alternative intervention. Probiotics or placebo were administered locally into the peri-implant sulcus and systemically as daily lozenges.	Both treatment protocols resulted in reductions in peri-implant plaque accumulation and bleeding on probing during follow-up. No significant differences in modified PI or the number of BOP-positive sites were observed between the probiotic and placebo treatments at any evaluation time point. Adjunctive local and systemic probiotic administration did not provide additional clinical benefits over PAPR + PDT alone for the evaluated peri-implant mucositis outcomes.	Not explicitly reported in the available data
Cláudio et al., 2024 [28]	51 patients with poorly controlled type 2 diabetes mellitus and residual periodontal pockets (PD ≥ 4 mm with BoP) were randomized into three groups (n = 17 each): SI alone, SI combined with BM, or SI combined with the BM and aPDT. Clinical, glycemic, and immunological outcomes were assessed at baseline, 90 days, and 180 days.	All treatment groups showed improvements in periodontal clinical parameters during follow-up. The BM + aPDT group showed a greater reduction in the percentage of residual periodontal pockets with PD ≥4 mm and BOP compared with subgingival instrumentation alone, particularly at 90 days. The BM + aPDT group demonstrated a reduction in the pro-inflammatory cytokine TNF-α at 90 days, whereas no consistent significant differences were observed for the other evaluated inflammatory markers. No significant between-group differences were observed in glycemic control, as assessed by HbA1c levels. As BM (oxygen-releasing gel) was not evaluated in a conventional aPDT-only comparator arm, the independent contribution of oxygen enhancement to the aPDT component cannot be isolated from this design; findings are reported as effects of the combined BM + aPDT protocol.	Not explicitly reported in the available data
Panhóca et al., 2016 [29]	24 orthodontic patients undergoing fixed appliance therapy were randomized into four parallel groups: blue light irradiation alone, curcumin-mediated aPDT, curcumin-mediated aPDT with 0.1% SDS, or CHX mouthwash. Saliva samples were collected at baseline, after rinsing, and following aPDT for microbiological analysis. Outcomes reflect salivary *S. mutans* measurements rather than site-specific plaque/biofilm sampling.	Curcumin-mediated aPDT and SDS-containing aPDT significantly reduced salivary *S. mutans* viability. SDS-containing aPDT produced a greater numerical reduction in salivary *S. mutans* than curcumin-mediated aPDT alone. CHX showed the greatest numerical antimicrobial effect; however, no significant difference was observed between CHX and SDS-containing aPDT. Overall, SDS-containing aPDT showed favorable antimicrobial activity, although a significant direct advantage over curcumin-mediated aPDT alone was not reported.	Not explicitly reported in the available data
Vakaki et al., 2025 [30]	60 adult smokers with Stage III or IV periodontitis were randomized into two parallel groups (n = 30 each). The control group received conventional NSPT alone. The test group received NSPT supplemented with daily home-use dual-light aPDT for 4 months. Clinical and biomarker outcomes were assessed at baseline, 2 weeks, 4 months, and 6 months.	BoP decreased significantly from baseline in both groups; however, no significant between-group differences were observed at 4 or 6 months. PD and CAL improved significantly over time in both groups, with no additional clinical benefit of adjunctive dual-light aPDT compared with NSPT alone. Plaque indices generally improved during follow-up, with no significant differences between groups. aMMP-8 levels decreased over time in both groups, but the changes were not statistically significant.	Dual-light aPDT was generally well-tolerated; mild device-related discomfort and one minor, fully resolved papilla burn were reported.
Pakarinen et al., 2022 [31]	59 patients with periodontitis were randomized to receive either NSPT alone or NSPT supplemented with daily home-use dual-light aPDT. The intervention was continued for 3 months. This interim analysis included 51 patients in the final analysis, with clinical and microbiological outcomes assessed after 3 months.	Adjunctive home-use dual-light aPDT resulted in a greater reduction in BoP at 3 months compared with NSPT alone, with more patients achieving the BoP < 10% target. Both groups showed reductions in deep periodontal pockets; however, greater pocket reduction was observed with adjunctive dual-light aPDT, including a higher proportion of patients achieving ≥50% reduction. Plaque levels decreased significantly in the dual-light aPDT group, while no significant improvement was observed with NSPT alone.	Compliance with home-use dual-light aPDT was high, with no serious device-related adverse events; only mild transient sensations were reported.
Sudhakar et al., 2025 [32]	30 orthodontic patients undergoing fixed appliance therapy were randomized into three parallel groups (n = 10 each). Participants received weekly applications of 0.2% CHX varnish, riboflavin-mediated aPDT, or riboflavin-mediated aPDT combined with SDS. All interventions were administered once weekly for 4 weeks. Plaque samples were collected at baseline, after 30 days, and at 2-month follow-up for microbiological assessment of *S. mutans* and *L. acidophilus*, with biofilm viability additionally evaluated using CLSM. Outcomes were obtained from site-specific plaque/biofilm sampling rather than saliva.	Riboflavin-mediated aPDT significantly reduced plaque S. mutans and L. acidophilus counts (CFU-based), with partial persistence of the antimicrobial effect during follow-up. The addition of SDS did not enhance the CFU-based antimicrobial effect of riboflavin-mediated aPDT against *S. mutans* or *L. acidophilus*. CLSM biofilm-viability imaging (a distinct, non-CFU measure of antimicrobial effect) showed the lowest biofilm viability following riboflavin-mediated aPDT, whereas the SDS-enhanced protocol showed the highest residual viability, providing no evidence of additional benefit from SDS.	Not explicitly reported in the available data
Elsadek et al., 2023 [33]	75 patients with Stage III Grade B periodontitis were randomized into three parallel groups (n = 25 each). Participants received SRP combined with MB-loaded polyglycolic acid nanoparticles in *Aloe vera* gel, SRP combined with conventional MB-mediated aPDT, or SRP alone. Clinical and microbiological outcomes were assessed at baseline, 3 months, and 6 months.	All three treatment groups showed improvements in clinical periodontal parameters during follow-up—Conventional aPDT combined with SRP resulted in a greater reduction in BoP, whereas MB-loaded PGA nanoparticles in *Aloe vera* gel combined with SRP produced greater PD reduction and CAL gain compared with the other treatment approaches. Conventional aPDT combined with SRP reduced *T. forsythia* and *P. gingivalis*, while the nanoparticle-based formulation also reduced *T. forsythia* during follow-up. Overall, the MB-loaded nanoparticle formulation provided greater improvements in PD and CAL than conventional MB-mediated aPDT, although microbiological findings were less consistent, and the contribution of the *Aloe vera* vehicle relative to the nanoparticle itself could not be isolated (no aloe-vera-only comparator arm).	Not explicitly reported in the available data
Patyna et al., 2021 [34]	48 systemically healthy patients with Stage II or III, Grade B periodontitis were randomized into three parallel groups (n = 16 each). Participants received SD alone, SD combined with aPDT, or SD combined with aPDT and adjunctive probiotics (*Levilactobacillus brevis* and *Lactiplantibacillus plantarum*) administered locally and systemically for 3 months. Clinical and microbiological outcomes were assessed at baseline, 3 months, and 6 months.	All three treatment groups showed improvements in PPD and CAL, with no significant between-group differences at 3 or 6 months. Adjunctive probiotics combined with SD and aPDT resulted in greater reductions in BoP and gingival inflammation compared with treatment without probiotics. The probiotic-enhanced protocol also produced greater reductions in *P. gingivalis* and *T. forsythia* at 6 months, while no significant differences were observed for the other investigated periodontal pathogens.	No adverse events or complications were reported.
Lähteenmäki et al., 2025 [35]	40 patients with dental implants, diagnosed as peri-implant healthy, with peri-implant mucositis, or with peri-implantitis, were randomized into two parallel groups (n = 20 each). Participants received oral hygiene instructions either alone or supplemented with home-use dual-light aPDT for 4 weeks. Dual-light aPDT was performed once daily for the first 2 weeks and twice daily for the following 2 weeks. Clinical and biomarker outcomes were assessed at baseline, 2 weeks, and 4 weeks.	aMMP-8 levels decreased during treatment with adjunctive dual-light aPDT, whereas no corresponding reduction was observed in the control group. Plaque levels decreased in both groups, with no significant between-group difference. Adjunctive dual-light aPDT resulted in a marked reduction in BoP, while no corresponding improvement was observed in the control group. Reduction in the deepest peri-implant PPD occurred only in the dual-light aPDT group, although the between-group difference was not statistically significant.	Not explicitly reported in the available data
El Refai et al., 2025 [36]	24 patients with Stage II Grade B periodontitis were randomized into two parallel groups (n = 12 each). Participants received NSPT combined with repeated MB-mediated aPDT or the same treatment supplemented with daily oral vitamin D3. aPDT was performed at baseline and repeated weekly for three additional sessions, while vitamin D3 supplementation was continued for 6 weeks. Clinical outcomes were assessed at baseline, 4 weeks, and 6 weeks.	CAL improved in both groups, with a greater improvement following adjunctive vitamin D supplementation; the between-group difference favored the vitamin D group at 6 weeks. PI and GI decreased in both groups, with greater reductions in the vitamin D group at 4 and 6 weeks. Overall, adjunctive vitamin D supplementation was associated with greater short-term clinical improvement compared with NSPT and aPDT alone.	Not explicitly reported in the available data
Malgikar et al., 2016 [37]	24 patients with untreated chronic periodontitis participated in a split-mouth randomized controlled trial. Selected sites within each patient were randomized to SRP alone, SRP plus aPDT, or SRP plus aPDT plus adjunctive LLLT. Clinical outcomes were assessed at baseline, 1 month, 3 months, and 6 months.	All three treatment approaches resulted in improvements in clinical periodontal parameters during the 6-month follow-up. SRP combined with aPDT and LLLT produced greater PD reduction and CAL gain than SRP combined with aPDT or SRP alone. Overall, adjunctive LLLT provided additional clinical benefit when added to SRP and aPDT during the 6-month follow-up.	No postoperative pain, discomfort, or treatment-related complications were reported.

MB—methylene blue; MBS—methylene blue in a 0.25% sodium dodecyl sulfate surfactant vehicle; aPDT—antimicrobial photodynamic therapy; CFU/mL—colony-forming units per milliliter; SDS—sodium dodecyl sulfate; PPD—probing pocket depth; MD—mechanical debridement; PDT—photodynamic therapy; PGA/RF/AV + MD—mechanical debridement combined with poly-L-glycolic acid nanoparticles loaded with riboflavin and incorporated in *Aloe vera* gel; PDT + MD—antimicrobial photodynamic therapy with riboflavin as the photosensitizer combined with mechanical debridement; PI—plaque index; PD—probing depth; MBL—marginal bone level; BoP—bleeding on probing; PAPR—professionally administered plaque removal; PAPR + PDT—professionally administered plaque removal combined with photodynamic therapy; BM—subgingival instrumentation combined with an active oxygen-releasing gel containing sodium percarbonate; BM + aPDT—subgingival instrumentation combined with the oxygen-releasing gel and antimicrobial photodynamic therapy; TNF-α—tumor necrosis factor alpha; HbA1c—glycated hemoglobin; NSPT—non-surgical periodontal therapy; CAL—clinical attachment level; CHX—chlorhexidine; CLSM—confocal laser scanning microscopy; PGA—polyglycolic acid; PGA/MB/AV—*Aloe vera* gel containing polyglycolic acid nanoparticles loaded with methylene blue; SRP—scaling and root planing; SD—subgingival mechanical debridement; GI—gingival index; LLLT—low-level laser therapy.

**Table 5:** Characteristics of the aPDT system used.

Author and Year	Photosensitizer	PS Concentration (as Reported)	Approx. Molar Equivalent (Calculated) *	Light Source	Wavelength (nm)	Irradiation Parameters	Radiant Exposure (J/cm^2^) Calculable?
Alvarenga et al., 2019 [24]	Methylene blue	100 μM	100 μM	Diode laser	660	100 mW; 1, 3, or 5 min; irradiance 250 mW/cm^2^; energy 6, 18, or 30 J per point	Yes (explicitly reported)
Kassa et al., 2022 [25]	Methylene blue	100 μM	100 μM	Diode laser	660	100 mW; 2 min per site; irradiance 0.25 W/cm^2^; radiant exposure 30 J/cm^2^, continuous mode	Yes (explicitly reported)
Qamar et al., 2023 [26]	Riboflavin	0.1%	≈2657 μM (approx.; (initial solution)	Diode laser	670	280 mW; 60 s per site; irradiance 1.1 W/cm^2^	Yes (calculable: ≈66 J/cm^2^)
Mongardini et al., 2017 [27]	Toluidine blue O	0.1 mg/mL (0.01%)	≈327 μM (approx.)	LED lamp (FotoSan^®^)	630	10 s at each of six peri-implant sites	No (irradiance not reported)
Cláudio et al., 2024 [28]	Methylene blue	100 μg/mL	≈313 μM (approx.)	InGaAlP diode laser	660 ± 10	100 mW; 50 s; energy 5 J; radiant exposure 166 J/cm^2^; optical fiber output area 0.03 cm^2^	Yes (explicitly reported)
Panhóca et al., 2016 [29]	Curcumin	1 g/L	≈2715 μM (approx.)	LED (intra- and extraoral devices)	450 ± 10	Extraoral: 200 mW, 180 s; 80 mW/cm^2^, 36 J, 14 J/cm^2^. Intraoral: 1200 mW, 180 s; 472 mW/cm^2^, 216 J, 85 J/cm^2^	Yes (explicitly reported)
Vakaki et al., 2025 [30]	Indocyanine green	250 μg/mL	≈323 μM (approx.)	Dual-light LED (Lumoral^®^)	405 + 810	10 min per session	No (irradiance not reported)
Pakarinen et al., 2022 [31]	Indocyanine green	250 μg/mL	≈323 μM (approx.)	Dual-light LED (Lumoral^®^)	405 + 810	10 min; radiant exposure 30 J/cm^2^	Yes (explicitly reported)
Sudhakar et al., 2025 [32]	Riboflavin (as riboflavin-5-phosphate disodium salt)	0.5%	≈≈10,453 μM (approx.)	LED light	385–515	Irradiance 2000 mW/cm^2^; 180 s per surface; fluence 95 J/cm^2^	Yes (explicitly reported)
Elsadek et al., 2023 [33]	Methylene blue	0.005%	≈156 μM (approx.)	Diode laser	670	280 mW; irradiance 1.1 W/cm^2^; 60 s per site	Yes (calculable: ≈66 J/cm^2^)
Patyna et al., 2021 [34]	Toluidine blue O	0.1 mg/mL (0.01%)	≈327 μM (approx.)	LED (FotoSan^®^)	628	Irradiance 2000–4000 mW/cm^2^; 10 s per tooth surface	Yes (calculable range: ≈20–40 J/cm^2^)
Lähteenmäki et al., 2025 [35]	Indocyanine green	250 μg/mL	≈323 μM (approx.)	Dual-light LED (Lumoral^®^)	405 + 810	10 min; radiant exposure 40 J/cm^2^	Yes (explicitly reported)
El Refai et al., 2025 [36]	Methylene blue	2%	≈62,500 μM (approx.)	Blue halogen curing light	470	Irradiance 620 mW/cm^2^; 5 min irradiation; continuous mode	Yes (calculable: ≈186 J/cm^2^)
Malgikar et al., 2016 [37]	Methylene blue	1%	≈31,270 μM (approx.)	Diode laser	980	Pulsed mode; peak power 5 W; average power 1 W; 200 μs pulse/200 μs interval; 30–45 s per site	No (irradiated spot/fiber area not reported)

* Approximate molar concentrations (µM) were calculated from the mass concentrations reported in the original studies using the molecular weights of the respective photosensitizers. Percentage concentrations were assumed to be expressed as weight per volume (% *w*/*v*) where not otherwise specified. Calculated values were rounded to the nearest whole number. Abbreviations: PS—photosensitizer; LED—light-emitting diode; InGaAlP—indium gallium aluminum phosphide.

**Table 6:** Enhancement strategies used in antimicrobial photodynamic therapy in the included studies.

Author and Year	Type of Adjuvant	Enhancement Category	Proposed Mechanism
Alvarenga et al., 2019 [24]	Sodium dodecyl sulfate (0.25% SDS; surfactant vehicle)	Physicochemical (surfactant/permeabilizer)—direct enhancer	Increased membrane permeability and reduced photosensitizer aggregation
Kassa et al., 2022 [25]	Sodium dodecyl sulfate (0.25% SDS)	Physicochemical (surfactant/permeabilizer)—direct enhancer	Enhanced photosensitizer uptake and penetration
Qamar et al., 2023 [26]	Poly-L-glycolic acid nanoparticles incorporated into *Aloe vera* gel	Nanocarrier/bioactive vehicle—direct enhancer (nanocarrier) combined with a biologically active vehicle	Prolonged photosensitizer availability and enhanced interaction with target tissues and biofilm
Mongardini et al., 2017 [27]	*Lactiplantibacillus plantarum* and *Levilactobacillus brevis* probiotics administered locally and systemically	Biological (probiotic)—complementary adjunct	Potential effects on selected periodontal microorganisms and local host–microbe interactions
Cláudio et al., 2024 [28]	Active oxygen-releasing gel (sodium percarbonate) 0.4 mL	Oxygen-enhancing—direct enhancer	Increased oxygen availability for ROS generation
Panhóca et al., 2016 [29]	Sodium dodecyl sulfate (0.1% SDS)	Physicochemical (surfactant/permeabilizer)—direct enhancer	Enhanced photosensitizer penetration and bacterial membrane disruption
Vakaki et al., 2025 [30]	Dual-light irradiation (antimicrobial blue light + near-infrared aPDT)	Physical (light modulation)—direct enhancer	Combined endogenous chromophore activation by blue light and NIR-aPDT
Pakarinen et al., 2022 [31]	Dual-light irradiation (antimicrobial blue light + near-infrared aPDT)	Physical (light modulation)—direct enhancer	Combined endogenous chromophore activation by blue light and NIR-aPDT
Sudhakar et al., 2025 [32]	0.1% sodium dodecyl sulfate	Physicochemical (surfactant/permeabilizer)—direct enhancer	Intended to increase membrane permeability and photosensitizer diffusion; however, possible interference with riboflavin photochemical activity may reduce ROS-mediated killing
Elsadek et al., 2023 [33]	Polyglycolic acid nanoparticles loaded with methylene blue in *Aloe vera* gel (PGA/MB/AV)	Nanocarrier + natural bioactive vehicle—direct enhancer (nanocarrier) combined with a biologically active vehicle	Prolonged photosensitizer retention and controlled release, enhanced biofilm penetration, with potential additional bioactive effects of *Aloe vera*
Patyna et al., 2021 [34]	Probiotics administered locally and systemically (*Levilactobacillus brevis* and *Lactiplantibacillus plantarum*)	Biological (probiotic)—complementary adjunct	Potential effects on selected periodontal microorganisms and local host–microbe interactions
Lähteenmäki et al., 2025 [35]	Dual-light irradiation (antimicrobial blue light + near-infrared aPDT)	Physical (light modulation)—direct enhancer	Combined endogenous chromophore activation by blue light and NIR-aPDT
El Refai et al., 2025 [36]	Oral vitamin D3 supplementation (cholecalciferol, 5000 IU) administered for 6 weeks	Biological (host-modulating)—complementary adjunct	Potential modulation of host inflammatory responses and support of periodontal tissue healing
Malgikar et al., 2016 [37]	Low-level laser therapy (LLLT)	Physical (photobiomodulation)—complementary adjunct	Photobiomodulatory effects intended to support periodontal tissue healing and modulate inflammation

ROS—reactive oxygen species; SDS—sodium dodecyl sulfate; aPDT—antimicrobial photodynamic therapy; ICG—indocyanine green; PGA—polyglycolic acid; MB—methylene blue; AV—*Aloe vera*; LLLT—low-level laser therapy; NIR—near-infrared.

**Table 7:** Dental fields and biofilm-related conditions investigated in the included studies.

Author and Year	Dental Field	Biofilm-Related Disease/Condition
Alvarenga et al., 2019 [24]	Periodontology	Periodontitis
Kassa et al., 2022 [25]	Periodontology	Periodontitis
Qamar et al., 2023 [26]	Implant dentistry/Periodontology	Peri-implantitis
Mongardini et al., 2017 [27]	Implant dentistry/Periodontology	Peri-implant mucositis
Cláudio et al., 2024 [28]	Periodontology	Periodontitis
Panhóca et al., 2016 [29]	Oral microbiology/Preventive dentistry	Oral biofilm/dental plaque
Vakaki et al., 2025 [30]	Periodontology	Periodontitis
Pakarinen et al., 2022 [31]	Periodontology	Periodontitis
Sudhakar et al., 2025 [32]	Orthodontics	Orthodontic appliance-associated dental biofilm
Elsadek et al., 2023 [33]	Periodontology	Stage III, Grade B periodontitis
Patyna et al., 2021 [34]	Periodontology	Stage II-III, Grade B periodontitis
Lähteenmäki et al., 2025 [35]	Implant dentistry/Periodontology	Peri-implant health, peri-implant mucositis and peri-implantitis
El Refai et al., 2025 [36]	Periodontology	Stage II, Grade B periodontitis
Malgikar et al., 2016 [37]	Periodontology	Chronic periodontitis

No eligible randomized trial addressing an oral fungal biofilm-associated condition (e.g., oral candidiasis) met the review’s inclusion criteria; consequently, Table 7 and the synthesis in Section 3 are limited to bacterial biofilm-associated conditions despite the review question, as defined in Section 2.1, encompassing both bacterial and fungal disease.

**Table 8:** Geographic distribution of the included research.

Author and Year	Country
Alvarenga et al., 2019 [24]	Brazil
Kassa et al., 2022 [25]	Brazil
Qamar et al., 2023 [26]	Saudi Arabia
Mongardini et al., 2017 [27]	Italy
Cláudio et al., 2024 [28]	Brazil
Panhóca et al., 2016 [29]	Brazil
Vakaki et al., 2025 [30]	Greece
Pakarinen et al., 2022 [31]	Finland
Sudhakar et al., 2025 [32]	India
Elsadek et al., 2023 [33]	Saudi Arabia
Patyna et al., 2021 [34]	Germany
Lähteenmäki et al., 2025 [35]	Finland
El Refai et al., 2025 [36]	Egypt
Malgikar et al., 2016 [37]	India

## Data Availability

All data extracted and analyzed in this systematic review are provided in the manuscript.
